# Recent Evolution of a Maternally Acting Sex-Determining Supergene in a Fly with Single-Sex Broods

**DOI:** 10.1093/molbev/msad148

**Published:** 2023-06-23

**Authors:** Robert B Baird, John M Urban, Andrew J Mongue, Kamil S Jaron, Christina N Hodson, Malte Grewoldt, Simon H Martin, Laura Ross

**Affiliations:** Institute of Evolutionary Biology, University of Edinburgh, Edinburgh, United Kingdom; Department of Embryology, Carnegie Institution for Science, Howard Hughes Medical Institute Research Laboratories, Baltimore, MD, USA; Institute of Evolutionary Biology, University of Edinburgh, Edinburgh, United Kingdom; Tree of Life Programme, Wellcome Sanger Institute, Cambridge, United Kingdom; Department of Zoology, University of British Columbia, Vancouver, Canada; Department of Molecular Biology and Genetics, Aarhus University, Aarhus, Denmark; Institute of Evolutionary Biology, University of Edinburgh, Edinburgh, United Kingdom; Institute of Evolutionary Biology, University of Edinburgh, Edinburgh, United Kingdom

**Keywords:** sex determination, monogenic reproduction, supergene, evolutionary strata

## Abstract

Sex determination is a key developmental process, yet it is remarkably variable across the tree of life. The dipteran family Sciaridae exhibits one of the most unusual sex determination systems in which mothers control offspring sex through selective elimination of paternal X chromosomes. Whereas in some members of the family females produce mixed-sex broods, others such as the dark-winged fungus gnat *Bradysia coprophila* are monogenic, with females producing single-sex broods. Female-producing females were previously found to be heterozygous for a large X-linked paracentric inversion (X′), which is maternally inherited and absent from male-producing females. Here, we assembled and characterized the X′ sequence. As close sequence homology between the X and X′ made identification of the inversion challenging, we developed a k-mer–based approach to bin genomic reads before assembly. We confirmed that the inversion spans most of the X′ chromosome (∼55 Mb) and encodes ∼3,500 genes. Analysis of the divergence between the inversion and the homologous region of the X revealed that it originated very recently (<0.5 Ma). Surprisingly, we found that the X′ is more complex than previously thought and is likely to have undergone multiple rearrangements that have produced regions of varying ages, resembling a supergene composed of evolutionary strata. We found functional degradation of ∼7.3% of genes within the region of recombination suppression, but no evidence of accumulation of repetitive elements. Our findings provide an indication that sex-linked inversions are driving turnover of the strange sex determination system in this family of flies.

**Significance**

Some insects exhibit monogeny, a peculiar form of reproduction where sex is determined by the maternal genotype and mothers produce single-sex broods. In the fungus gnat *Bradysia coprophila*, this phenomenon is under the control of a huge X-linked nonrecombining region. Here, we identify the genomic sequence of this region and show that it resembles a supergene composed of multiple complex rearrangements, that it evolved recently, and that it has undergone degradation characteristic of evolving sex chromosomes. Our findings suggest that such sex-linked inversions may be driving the turnover of unusual reproductive strategies in fungus gnats.

## Introduction

Sex is an ancient feature shared by most eukaryotes, yet the sex determination systems regulating the development of males and females vary widely among animals (Beukeboom and Perrin 2014) and can evolve rapidly (Saunders and Veyrunes 2021). Why such a fundamental developmental process as sex determination is variable remains an outstanding question (Bachtrog et al. 2014). Insects include many examples of this diversity and are therefore an excellent model for understanding changes in sex determination systems. Although most insects have genetic sex determination mechanisms with distinct sex chromosomes, different chromosomes act as the sex chromosomes in different species, and species differ in whether males (XY and X0 systems) or females (ZW and Z0 systems) are the heterogametic sex, and in divergence between the sex chromosome pair (Bull 1983; Beukeboom and Perrin 2014). There are also examples of complete loss of sex chromosomes, where sex is linked to ploidy differences (e.g., haplodiploidy) or elimination or silencing of paternally derived chromosomes in males. Another remarkable case, where sex is determined chromosomally but in a way that fundamentally differs from the standard XY or ZW systems, is that of monogenic sex determination. Here, sex is determined by the genotype of the mother instead of that of the zygote: Mothers are genetically predetermined to produce either only male offspring or only female offspring. Monogenic sex determination has evolved in three clades of flies (Diptera): blowflies (Chrysomyinae, Ullerich 1958), gall midges (Cecidomyiidae, Benatti et al. 2010), and fungus gnats (Sciaridae, Metz 1938). Little is known about control of sex determination in blowflies (Scott et al. 2014). However, in the fungus gnat and gall midge species in which karyotypes have been characterized, monogeny appears to be associated with chromosomal inversions (Carson 1946; Crouse 1979; Benatti et al. 2010). None of these inversions has yet been characterized, and little is known about the nature and the molecular evolution of these regions. Neither the evolutionary history of monogeny nor how selection acts on sex determining regions that occur outside the context of conventional sex chromosomes is thus currently understood.

Suppression of recombination through chromosomal inversions occurs in some sex chromosomes (Vicoso 2019), and several scenarios can favor a lack of recombination (Wright et al. 2017; Connallon et al. 2018; Jay et al. 2022; Lenormand and Roze 2022). Prevailing theory posits that this process involves selection for suppressed recombination between the sex-determining locus on the Y or W chromosome, and sexually antagonistic alleles maintained polymorphically at partially sex-linked loci, potentially encompassing increasingly large portions of the sex chromosome in a stepwise process (Charlesworth and Charlesworth 1980; Rice 1987; Charlesworth et al. 2005). However, several alternative hypotheses have recently been proposed, including a role for local adaptation (Connallon et al. 2018), regulatory evolution (Lenormand and Roze 2022), and the buildup of deleterious mutations (Jay et al. 2022). Y- or W-linked inversions may create regions that never or rarely recombine with their homologous X- or Z-linked regions. This creates sex-specific transmission and ensures that the affected regions are always heterozygous, unlike autosomal inversions. Such regions are likely to accumulate adaptive mutations specific to one sex or the other (Connallon et al. 2018). If the region completely fails to recombine, it is liable to accumulate deleterious mutations and transposable elements (TEs) (Felsenstein 1974). As a result, the nonrecombining Y or W chromosomes undergo functional degradation (Bachtrog et al. 2008) and become a reservoir for repetitive sequences (Chalopin et al. 2015).

**Fig. 1.**
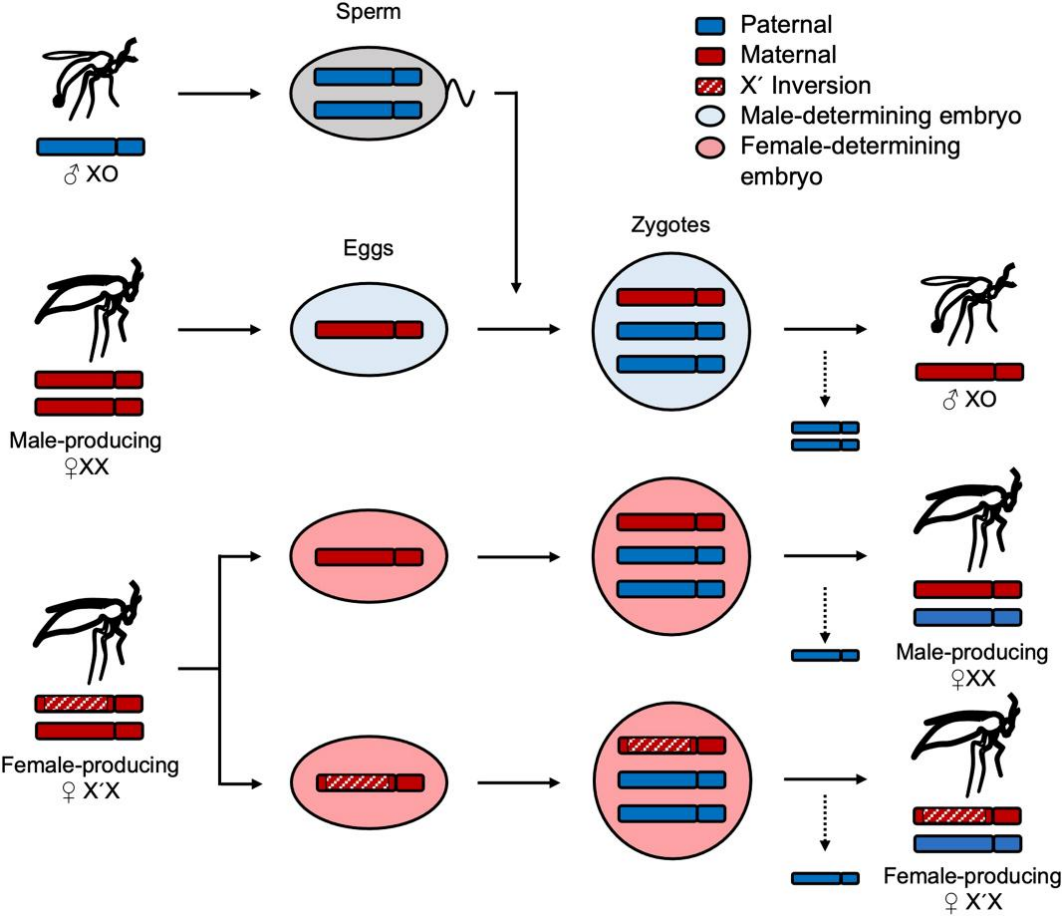
Sex determination and X chromosome inheritance in *B. coprophila*. While oogenesis is regular, sperm receive two X copies due to X nondisjunction. The mother's genotype determines offspring sex: All zygotes begin with three X chromosomes and lose either one or two paternal X chromosomes via targeted paternal genome elimination, resulting in female and male development respectively. XX females produce only sons whereas females heterozygous for the X′ (X′X) produce only daughters.

In the present study, we investigated a female-limited, nonrecombining X-linked inversion associated with monogeny in the fungus gnat *B.* (*Sciara*) *coprophila.* This species has been studied extensively since the 1920s (Metz 1938) and has a complex chromosome inheritance system (fig. 1). Like all members of Sciaridae, it reproduces through paternal genome elimination, where males fail to transmit paternally derived chromosomes to their offspring as they undergo several rounds of maternally controlled chromosome elimination targeting the paternal genome (Metz 1938; Gerbi 2022). In all studied members of the Sciaridae, the somatic cells of males have an X0 karyotype, while those of females are XX. However, sex is determined by maternally controlled X elimination during early embryogenesis, rather than X inheritance. All zygotes begin with three X chromosomes, one inherited from the mother and two from the father—the result of aberrant spermatogenesis involving the nondisjunction of the sister chromatids in the second meiotic division. During the seventh to ninth embryonic cleavage divisions, either one or both paternal X chromosomes are eliminated from somatic cells, resulting in the zygotes developing into females (XX) or males (X0), respectively. The eliminated X chromosomes fail to divide at anaphase and are left behind on the metaphase plate (DuBois 1933). Germ cells in both sexes eliminate a single paternal X during a resting stage later in development.

In *B. coprophila* and many other Sciaridae, females are monogenic and produce single-sex progenies. Nonmonogenic sciarids are “digenic” and produce mixed-sex broods, although both monogenic and digenic species determine sex through X chromosome elimination. Both reproductive strategies occur in multiple Sciaridae genera (Metz 1938), though their evolutionary relationship to one another remains unclear. Early cytological observations suggested that two monogenic species, *B. coprophila* and *Bradysia impatiens*, possess single long inversions spanning most of the X chromosome (henceforth the inverted chromosome is denoted by X′), for which female-producing females are heterozygous (Carson 1946; Crouse 1979). Polytene chromosome staining indicates that such inversions are absent in digenic species (McCarthy 1945a, 1945b) as well as in at least one species exhibiting mixed reproductive strategies (Rocha and Perondini 2000). Through a series of cytogenetic studies, Crouse (1979) deduced the structures of the chromosomes in *B. coprophila* and demonstrated that the X′ inversion is paracentric and spans most of the length of the chromosome, leaving the two ends of the chromosome, which still synapse with the X, noninverted. The genome sequence of *B. coprophila*, with all three autosomes and the X chromosome, has recently been published (Urban et al. 2021; 2022), though the sequence and precise nature of the X′ inversion remain unknown as the reference genome was generated from X0 males, which lack the X′.

Here, we have shown through comparative analysis of X and X′ chromosomes in *B. coprophila* that the structure of the X′ is likely more complex than previously thought. Rather than a single paracentric inversion, we found that it resembles a supergene composed of multiple linked inversions that all emerged <0.5 Ma. Our finding that the X′ is young is intriguing given that monogeny is shared by multiple Sciaridae genera (Metz 1938) and suggests that inversions may drive the turnover of reproductive strategies in this family. We used a novel process of k-mer binning to assign short reads to chromosomes prior to assembly, allowing assembly of ∼55 Mb corresponding to X′ supergene sequence despite its high sequence similarity to the ancestral X chromosome. With assembly and annotation of the X′, we compared patterns of evolution between the two homologous sequences and found that the supergene shows some early signs of degradation characteristic of other norecombining sex chromosomes and supergenes. We discuss the implications of our findings for disentangling the evolutionary relationship between the strange genetic properties of sciarid flies and in light of the evolution of sex chromosomes and sex-linked adaptive inversions.

## Results

### X-X′ Divergence Reveals Recent Evolution and Stratification of the X′ Chromosome

We set out to identify the breakpoints of the long paracentric inversion previously described in the literature. The size of the X chromosome in *B. coprophila* is estimated as 50–67 Mb (Gabrusewycz-Garcia 1964; Rasch 2006; Urban et al. 2021; Hodson et al. 2022), and the X′ inversion spans almost the entire chromosome length (Crouse 1979). We therefore expected the inversion to be slightly shorter than the X. We produced whole-genome sequencing Illumina libraries from X0, XX, and X′X individuals, which when aligned against the recently updated chromosome-scale reference genome that contains sequences for chromosomes X, II, III, and IV, but not X′ (Bcop_v2, Urban et al. 2022), resulted in mapping rates of 93.58%, 96.68%, and 96.34%, respectively. That the X′X libraries have approximately the same mapping rate as the XX libraries indicate there is high enough sequence identity between the X and X′ to reliably call structural (SVs) and single-nucleotide variants (SNVs). We found that the lower mapping rate of the X0 reads was explained mostly by a higher microbial content in those libraries (supplementary Text S1, Supplementary Material online).

**Fig. 2.**
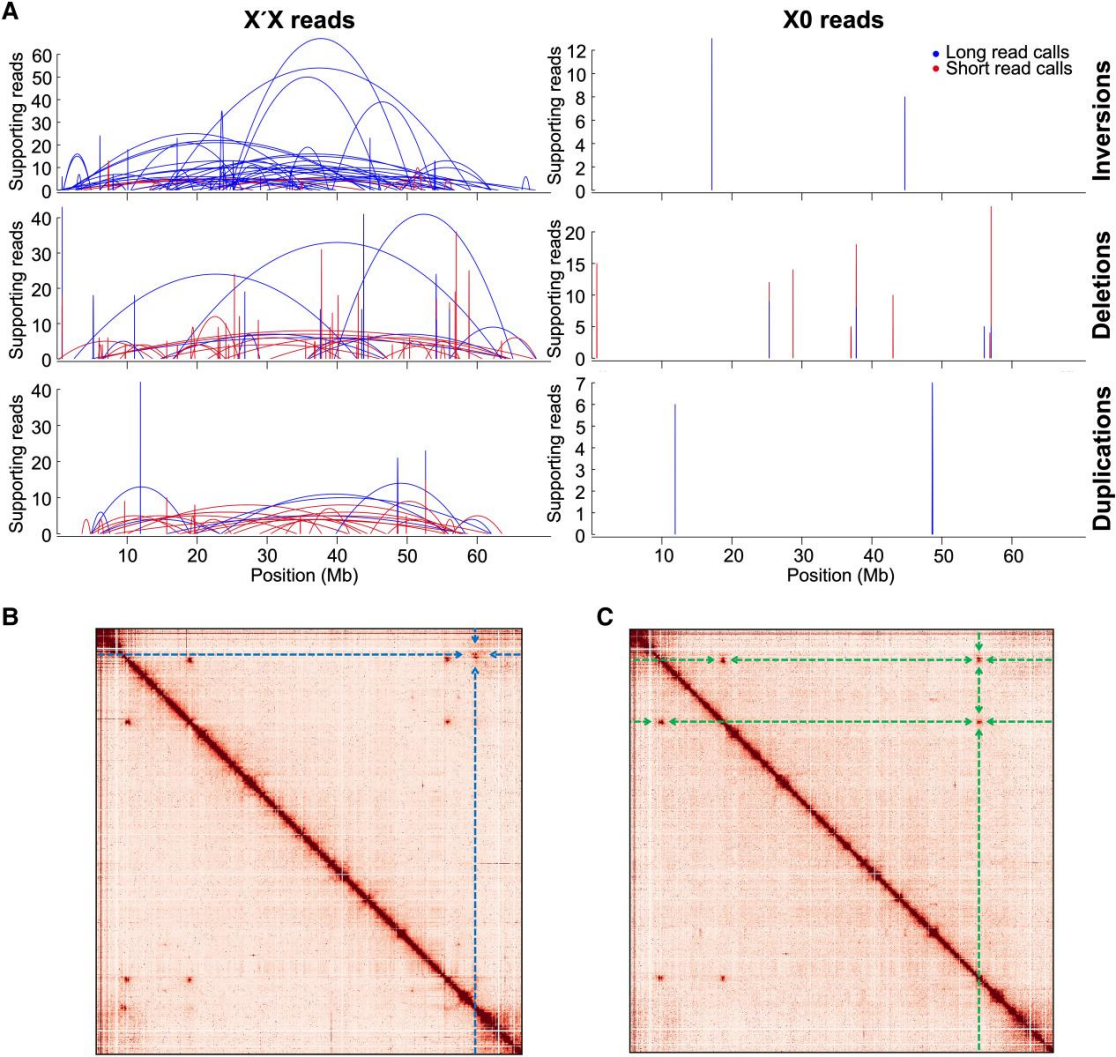
(*A*) SV calls from the X′X genotype are enriched across the X chromosome compared with calls from the X0 genotype, indicating more complex rearrangements for the X′ than may be explained by a single paracentric inversion. Start and end positions of SVs are shown with arcs. Only SVs supported by at least four reads and with spans >10 kb are shown. (*B*) HiC contact heatmap across the X chromosome for reads from the X′X genotype, as well as (*C*) for the X0 genotype. Contact showing the two main breakpoints is highlighted by dashed lines in (*B*). Repeats present in both heatmaps are highlighted by dashed lines in (*C*).

**Fig. 3.**
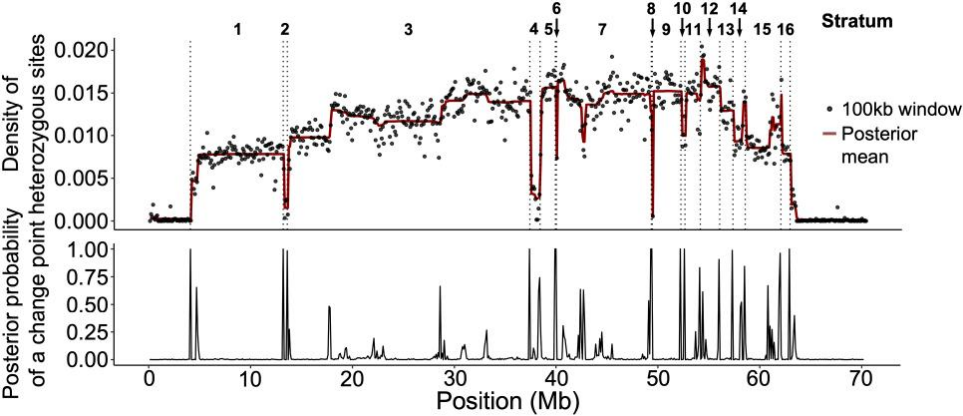
Upper panel: the distribution of variant sites between the X and X′, obtained from alignments of X′X reads to the X chromosome, along which posterior means were calculated. Lower panel: the probability of point changes between posterior means were used to delineate putative evolutionary strata. Putative breakpoints between strata are shown as dotted lines in the upper panel.

In an attempt to identify the breakpoints of the long paracentric inversion on the X′, we searched for SVs that could be attributed to the X′ using both Illumina short-read and PacBio long-read alignments from X′X samples, using XX and X0 samples as a control. However, this analysis demonstrated that, in the X′X samples, the region of the X chromosome corresponding to the inverted region on the X′ is highly enriched for discordant paired-read and split long-read alignments that yield long, overlapping SV signals. We interpreted the entangled and contradictory nature of many individual SV calls as suggesting the presence of multiple complex rearrangements and transpositions throughout the region rather than one single paracentric inversion (fig. 2*A*, table 1, and supplementary fig. S1 and table S6, Supplementary Material online). In contrast, HiC reads from X′X and X0 genotypes mapped against the X chromosome clearly revealed the two “main” breakpoints observed cytologically, as well as three repeat regions that likely correspond to folds in the X chromosome (Crouse 1979), but did not clearly show additional breakpoints along the chromosome (fig. 2*B* and *C*). Nonetheless, SNV calls from alignments of X′X Illumina reads to the X chromosome revealed multiple distinct segments of the inversion with different SNV densities, again suggesting that multiple adjacent and/or nested inversions may have occurred at different times, perhaps in a stepwise fashion (fig. 3). We used these SNV calls to delineate putative evolutionary strata using a change-point analysis, and we estimated divergence for each stratum (fig. 3, table 2, and supplementary table S1, Supplementary Material online). We found that the region of recombination suppression spans between ∼4.1 and 62.9 Mb on the 67.2-Mb X chromosome. All strata were predicted to have emerged <0.5 Ma. *Dxy* values calculated from all sites across the chromosome region were 0.0006 for the youngest stratum and 0.0159 for the oldest stratum, corresponding to divergence in years of 0.008 and 0.107 Ma, respectively (assuming a similar mutation rate to *Drosophila*; see Materials and Methods). Notably, some of the youngest strata had exceptionally low divergence. Estimates for neutrally evolving (synonymous) genic sites ranged from 0.099 to 0.335 Ma for the youngest and oldest strata, respectively. Taken together, our findings suggest that a stepwise set of genomic rearrangements formed the X′ chromosome; we therefore set out to target the X′ sequence for de novo assembly.

**Table 1.** Number of Each Type of SV Call from X0 and X′X Alignments to the X Chromosome.

Support	SV	X0 Genotype	X′X Genotype
Short reads (Illumina)	Deletion	20	2,697
	Duplication	1	56
	Inversion	0	29
Long reads (PacBio)	Deletion	9	4,037
	Duplication	3	57
	Inversion	11	267

**Table 2.** Divergence Estimates for Putative X′ Strata in Millions of Years.

Stratum	Length (Mb)	N Homologs	*Dxy*	*Dxy* Estimate Midpoint	Neutral Estimate Midpoint	This Study's Lowest Estimate	This Study's Highest Estimate
S1	9.00	326	0.0075	0.099	0.147	0.050	0.219
S2	0.50	15	0.0040	0.053	0.098	0.027	0.145
S3	23.80	904	0.0123	0.162	0.221	0.083	0.329
S4	1.00	35	0.0039	0.052	0.067	0.026	0.099
S5	1.65	55	0.0151	0.198	0.292	0.101	0.434
S6	0.10	2	0.0074	0.097	0.099	0.049	0.147
S7	9.40	350	0.0145	0.191	0.290	0.097	0.432
S8	0.10	0	0.0006	0.008	NA	0.004	0.011
S9	2.55	62	0.0153	0.200	0.318	0.102	0.474
S10	0.40	22	0.0122	0.161	0.233	0.082	0.346
S11	1.50	50	0.0145	0.191	0.277	0.097	0.412
S12	1.90	96	0.0159	0.210	0.335	0.107	0.499
S13	1.30	90	0.0136	0.179	0.260	0.091	0.386
S14	1.20	36	0.0104	0.137	0.286	0.070	0.425
S15	3.50	97	0.0096	0.127	0.147	0.065	0.218
S16	0.95	54	0.0093	0.122	0.200	0.062	0.297
**Total**	**58**.**85**	**2,194**	**0**.**0054**	**0**.**136**	**0**.**218**	**0**.**070**	**0**.**304**

Note.—*Dxy* estimates are calculated from the density of heterozygous sites within each stratum; neutral estimates are calculated from the proportion of synonymous variants in single-copy X-X′ homologs within each stratum. Midpoints are means between ages calculated using different estimates of mutation rates and generation times.

### De Novo Assembly of the X′ Sequence

**Fig. 4.**
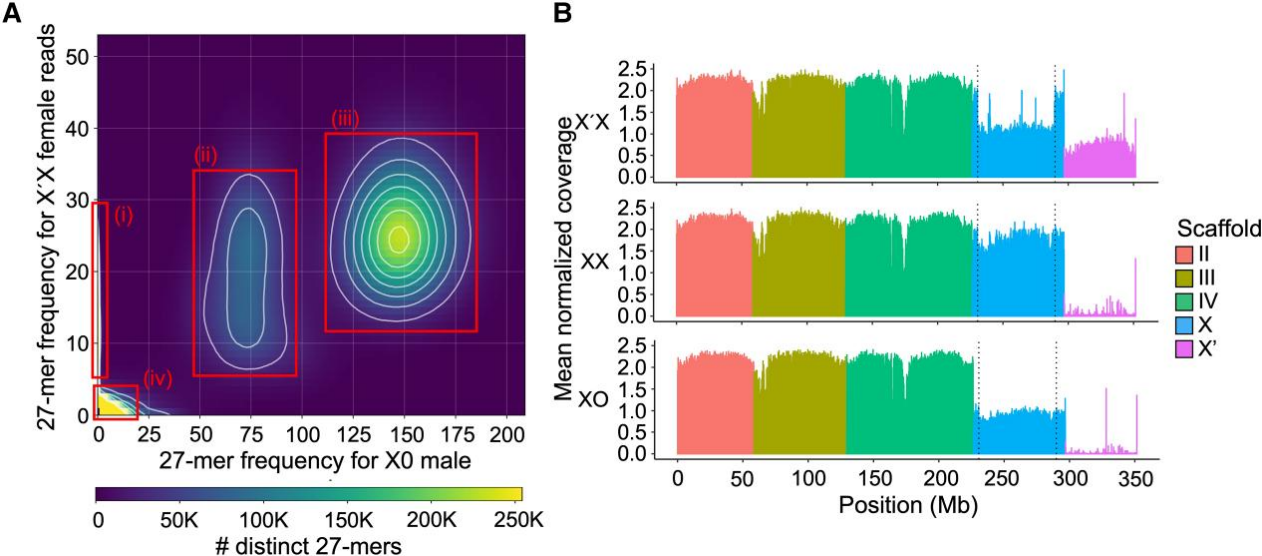
(*A*) A 27-mer frequency heatmap between Illumina reads from X0 versus X′X flies; 27-mers form dense clouds based on k-mer frequency, which reflects ploidy: 1) 27-mers specific to X′X are assigned as putative X′-specific 27-mers; 2) 27-mers haploid in X0 but diploid in X′X are likely those belonging to the X and the portion of X′ shared by both chromosomes; 3) 27-mers diploid in both sexes are likely those belonging to autosomes; and 4) 27-mers containing read errors cluster around the origin. (*B*) Mean normalized per-based genomic coverage across 100-kb windows of all autosomes, the X and the X′-supergene sequence, for reads from pooled individuals of each genotype: X′X (top), XX (middle), and X0 (bottom). The main breakpoints of the X′ (dotted lines) are clearly visible in coverage from X′X reads.

We attempted assembly of PacBio reads from X′X individuals, followed by chromosome assignment of scaffolds using sex differences in read depth across the genome (supplementary Text S2 and fig. S2, Supplementary Material online). This yielded a genome size of only 291 Mb, which was comparable with the size of the male (X0) genome (Urban et al. 2021). Moreover, we were able to assign only ∼3.6 Mb as putative X′ sequence (supplementary table S2, Supplementary Material online). High sequence identity between reads originating from the X and X′ chromosomes was likely leading to their collapsing together upon assembly. To overcome this, we used a process akin to haplotype resolution of diploid sequences by trio binning (Koren et al. 2018). Our approach utilizes differences in k-mer frequencies in Illumina reads between sexes to assign them to chromosomes prior to assembly. Taking advantage of high homozygosity due to over a century of inbreeding (Metz 1938) and the fact that X′ is limited to X′X individuals, we assigned k-mers specific to X′X female reads as likely to belong to the X′ (fig. 4*A*). We used these k-mers to extract the short reads from the X′X data set as putative X′-specific reads. In contrast to long reads, which have a high likelihood of false k-mer matches due to high sequencing error rates, we found that short reads (75–150 bp) can effectively be binned with k-mers due to their low error rate and short length (supplementary Text S3, Supplementary Material online).

The resulting putatively X′-specific Illumina reads assembled into 61.7 Mb across 42,564 contigs, with an N50 of 10 kb and a largest contig length of 87 kb (supplementary fig. S3, Supplementary Material online). We performed reference-based scaffolding of these contigs, using the regular X chromosome scaffold as a reference (Urban et al. 2022), to produce a single scaffold corresponding to the X′ supergene. To gap-fill and polish the X′ scaffold, we combined it with the remaining chromosomes II, III, IV, and X (Urban et al. 2022) and then used PacBio reads from X′X individuals competitively mapped against all chromosomes to fill some remaining gaps (supplementary fig. S4, Supplementary Material online) and Illumina reads from X′X individuals to polish the final assembly. The resulting ∼55-Mb scaffold is the first model of the X′ sequence contained within the long paracentric inversion breakpoints defined by Crouse (1979) (table 3). Due to using the uninverted X as a reference for scaffolding the X′ contigs, this scaffold may be an inaccurate structural representation of the X′ chromosome; thus, we did not attempt to use this assembly to infer information about the structure of the X′. However, alignment of Illumina reads from all three genotypes (X0, XX, and X′X) to all chromosomes strongly supports its correspondence to the X′ chromosome (fig. 4*B*). As expected, we observed that 1) X′X individuals had haploid coverage (1n) across the X′ sequence and the corresponding inverted region of the X chromosome compared with the autosomes, 2) XX and X0 individuals had very low coverage of the X′, and 3) XX females had relatively equal coverage across the X and autosomes in XX females (fig. 4*B*).

**Table 3.** Assembly Statistics for the *B. coprophila* Chromosome-Level Genome, Also Showing the X′ Supergene Sequence as well as the Portion of the X Homologous to the Supergene.

Chromosome	Size (Mb)	Size (excluding Ns, Mb)	Gaps	Predicted Genes	Gene Density	TE Density
II	58.34	57.99	26	3,968	10.40%	26.06%
III	71.05	69.01	101	5,919	12.68%	26.92%
IV	97.08	95.49	90	6,730	10.57%	28.30%
X	70.51	67.23	187	3,881	8.69%	23.06%
X (homologous portion)	58.80	56.87	136	3,429	8.54%	17.32%
X′-supergene	54.74	54.50	3,486	3,470	7.66%	10.51%
Total (excluding X′)	296.98	289.71	404	20,498	10.60%	26.31%
Total	351.73	344.11	3,194	23,968	10.14%	23.81%

Note.—Predicted genes shown are those predicted and annotated in this study. Gene and TE density were calculated from total gene and TE base pairs as a proportion of the length of each chromosome, excluding N bases.

### Functional Degradation of the X′ Chromosome

We identified 3,470 protein-coding genes totaling 4.2 Mb, that is, 7.7% of the 54.7-Mb X′ supergene scaffold. The portion of the X chromosome homologous to the supergene spans 58.8 Mb and contains 3,429 genes totaling 4.9 Mb, that is, 8.3% of the region. Thus, the proportion of the chromosome corresponding to coding sequence is slightly but not significantly lower on the X′ supergene relative to the homologous X region (*χ*^2^ = 0.00027, *df* = 1, *P* = 0.9869). We also found both the X′ and X to have lower gene densities than the autosomes. The difference between gene densities of the X′ and X may reflect an increase in noncoding DNA within the supergene or, alternatively, may be a consequence of the assembled X′ sequence having more gaps than the X chromosome sequence (table 3). We identified 2,321 single-copy homologs between the X′ and X. A further 527 genes from the X′ and 679 genes from the X across 296 orthologous groups (OGs) were categorized as duplicates. In 64 duplicate OGs, the X′ and X chromosomes had the same number of gene copies. Of the remaining duplicate OGs, 162 had more copies on the X compared with the X′, while 70 had more copies on the X′. These may be due to X- and X′-specific duplication events or ancestral duplication and subsequent loss in one or the other chromosome. Unlike X mutations, X′ mutations should not be purged by recombination; thus, deletions and duplications on the X′ are the more likely explanation. We also found that 622 genes across 603 OGs were specific to the X′ and that 429 (across 359 OGs) were specific to the X. Gain of novel genes and whole-gene deletions from the X′ are possible explanations for these finding, although sequence divergence, misassemblies, or gaps may have also led to homologs not being found.

**Fig. 5.**
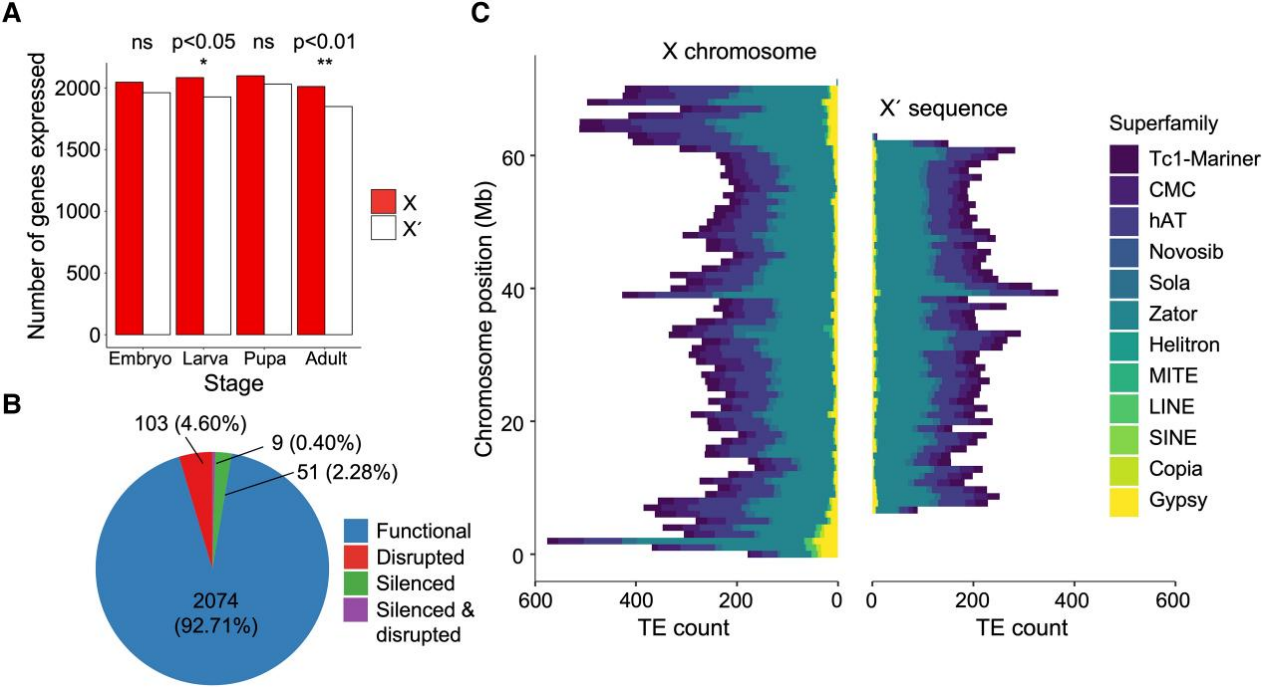
(*A*) Number of single-copy orthologous genes expressed at four developmental stages in females. Significantly more genes expressed from the X compared with the X′ chromosome in larvae and adults; other stages were nonsignificant (ns). (*B*) Proportion of functional versus nonfunctional X′-linked genes that have functional X homologs. (*C*) TE counts in 1-Mb bins distributed on the X chromosome and within the X′ supergene sequence.

We investigated the possibility that the X′ has undergone patterns of degeneration similar to other nonrecombining sex chromosomes by analyzing functional degradation of genes and accumulation of repetitive elements. Of the 2,321 single-copy gene OGs, we found that 123 (5.3%) contain X′-specific mutations that are likely to compromise gene function (including frameshift and/or gain or loss of stop or start codon mutations, supplementary table S3, Supplementary Material online). We further analyzed expression of single-copy homologs and found that fewer X′ genes are transcribed compared with their X-linked homologs: Across four life stages in females, 2,191 X′ homologs are expressed compared with 2,237 X homologs, although only the larval (*χ*^2^ = 6.07, *df* = 1, *P* = 0.014) and adult (*χ*^2^ = 6.80, *df* = 1, *P* = 0.009) stages had significantly fewer X′ copies expressed (fig. 5*A*). Overall, 7.3% of genes were classified as either silenced, disrupted, or both (fig. 5*B*).

The fly stock used in this study was derived from a laboratory stock maintained by Metz (1938), in which X′X females carry an X′-linked, irradiation-induced mutation, *Wavy*, which alters wing phenotype. As such, it is worth noting that this mutation may cause estimates of degradation to differ from wild-type flies, though its molecular nature is unknown. Among the genes classified as degraded, we identified several with functions in wing development, including the wing polarity protein STAN and the “held out wings” protein HOW, which are both required for regular wing development in *Drosophila* (Zaffran et al. 1997; Adler 2012) and may serve as candidates for the *Wavy* mutation. Since X′X females eliminate one rather than two X chromosomes from their embryos to produce only daughters, we may expect that this results from the silence or disruption of a maternal-effect X-linked gene on the X′ chromosome, which is somehow involved in the control of chromosome elimination. Among the genes classified as degraded, we found several candidates involved in chromatin regulation, chromosome segregation, and cohesion (supplementary Text S4 and table S4, Supplementary Material online).

We also checked X′X females for evidence of dosage compensation (DC) of X-linked genes that have corresponding degraded X′ homologs. We expected that if these genes were dosage compensated, they would be upregulated in X′X females to match the expression of those genes in XX females where both copies are functional. We compared expression of X-linked genes in X′X and XX female samples (supplementary fig. S5, Supplementary Material online). In total, only four X-linked genes were significantly upregulated in the X′X females (supplementary table S5, Supplementary Material online), none of which were genes that we classified as pseudogenized. Thus, we found no evidence of DC of degraded genes.

We also analyzed TE content across the genome. We found that TE density was lower on the sex chromosomes compared with the autosomes (table 3), but the difference was nonsignificant (*χ*^2^ = 0.0065, *df* = 1, *P* = 0.936). We did not find an enrichment of repetitive sequences on the X′; TE density within the X′-specific sequence was nonsignificantly lower (10.51%) compared with the homologous portion of the X (17.32%, *χ*^2^ = 0.060, *df* = 1, *P* = 0.807). The fact that the X′ sequence was assembled with short reads may have resulted in limited power to detect repetitive sequences compared with the X from the reference genome. Our analyses comparing structural differences between the X and X′ suggested that repetitive sequences, such as TEs, may have different or additional locations within the X′ sequence not present on the X (fig. 2*A*, table 1, and supplementary fig. S1 and table S6, Supplementary Material online). However, the distribution of TEs and TE superfamilies across our assembled X′ sequence appeared to be similar to that of the same chromosome region on the X (fig. 5*C*).

## Discussion

Recently evolved sex chromosomes can provide crucial insights into understanding the evolution and turnover of sex chromosomes. *Bradysia coprophila* is particularly unusual in that it exhibits a major transition from an X0-like system to one not unlike a ZW system, though only half of females are heterogametic and the sex chromosome is maternally acting rather than acting in the zygote. Thus, *Bradysia* offers a chance to study the early differentiation between nonrecombining regions of sex chromosomes in a unique evolutionary context. Our analysis and assembly of the ∼55-Mb X′ sequence revealed that it evolved very recently (<0.5 Ma), that it is more complex in structure than previously thought, and that it is beginning to undergo functional degradation characteristic of classical sex chromosome evolution.

### The X′ Chromosome Evolved Recently and Shows Signs of Degradation

In insects, emergences and turnovers of sex chromosomes are common (Vicoso and Bachtrog 2015). *Drosophila* neo-Y chromosomes are among the most well-studied cases. In *Drosophila*, males are achiasmatic, so Y-fused autosomes become instantly sex linked and nonrecombining. In the absence of recombination, deleterious mutations and TEs accumulate irreversibly because offspring inherit the full mutational load of their parents (Muller 1964). Lack of recombination further leads to hitchhiking effects of deleterious mutations, where beneficial mutations arising on the sex-limited chromosome spread to fixation and carry with them linked mildly deleterious loci (Maynard-Smith and Haigh 1974; Rice 1987). The inevitable result of such processes is functional degradation of the nonrecombining chromosomes. Studies of neo-sex chromosome evolution in *Drosophila* have indeed found that significant degeneration occurs rapidly: In *Drosophila pseudoobscura*, the neo-Y evolved ∼15 Ma and has very few genes remaining (Carvalho and Clark 2005; Mahajan and Bachtrog 2017). The *Drosophila miranda* neo-Y has undergone ∼4-fold expansion due to TE accumulation, and 40% of the ancestral genes have become pseudogenized in only ∼1.5 My of evolution (Bachtrog 2006; Bachtrog et al. 2008; Mahajan et al. 2018). The younger *Drosophila busckii* and *Drosophila americana* neo-Ys evolved ∼0.8 and <0.47 Ma and have 60% and 22% pseudogenized genes, respectively (Vieira et al. 2003; Zhou and Bachtrog 2015; Nozawa et al. 2021). Our finding that ∼7.3% of genes on the <0.5-Ma *B. coprophila* X′ chromosome are pseudogenized is consistent with this expected trajectory of sex chromosome evolution. Moreover, because the chromosome is present in only half of females, its effective population size is half that of other sex-limited chromosomes. As such, it should exhibit an accelerated rate of decay as the effects of drift should increase the rate of evolution further at sites under purifying selection (Charlesworth et al. 1987), though the effective population size of the sex chromosomes will depend on the relative reproductive success of males and females (Vicoso and Charlesworth 2009). To explore the potential effects of drift on sex-limited chromosome degeneration further, it will be essential to compare the X′ chromosomes of other Sciaridae species, which may have evolved independently and at different times, such as that of *B. impatiens* (see below, Carson 1946).

Sex chromosome degeneration is sometimes accompanied by the evolution of DC mechanisms to reestablish diploid expression of the X chromosome in males (Ohno 1967). In the ancestral *Drosophila* X chromosome, this is achieved by hypertranscription of X-linked genes in males (Schulze and Wallrath 2007). The neo-X chromosomes of various *Drosophila* species have achieved DC via transposon-mediated cooption of DC machinery, though the younger neo-Xs are yet to achieve global DC (Marín et al. 1996; Ellison and Bachtrog 2013; Zhou et al. 2013). In *B. coprophila*, there is evidence for DC in X0 males through upregulation of X expression (Urban et al. 2021). As for the X′X females, we found no evidence that X-linked genes with degraded X′ homologs show DC in X′X females. Given the young age of the X′, there may not have been sufficient time for the establishment of DC mechanisms to compensate for degraded X′ genes. Furthermore, because the X′ chromosome is present in only half of females, conflict between XX and X′X females over gene expression may hinder the evolution of DC.

Accumulation of repetitive sequences occurs rapidly in recently evolved sex chromosomes (Chalopin et al. 2015). The neo-Y chromosome of *D. miranda* has undergone massive TE accumulation and has expanded significantly as a result (Bachtrog et al. 2008; Mahajan et al. 2018), though the repetitive landscape of younger (<0.5 Ma) *Drosophila* neo-Ys (Nozawa et al. 2021) has not been analyzed and thus TE accumulation in nonrecombining regions over these shorter timescales is unclear. Despite the recent divergence between X and X′ in *B. coprophila*, one would expect higher TE content within the X′. Differences in TE accumulation may be affected by TE content: The proportion of DNA transposons relative to retrotransposons varies widely between lineages. We find that *B. coprophila*, similar to the *Musca*, *Aedes*, and *Culex* genera, has a higher proportion of DNA transposons than *Drosophila* (Petersen et al. 2019). Compared with the cut-and-paste mechanisms of DNA transposons, the copy-and-paste mechanisms of retrotransposons may lend themselves to more rapid accumulation (Kim et al. 2012). However, our approach using X′-specific k-mers to assemble the X′ may result in failure to assemble repetitive sequences that are shared by other chromosomes, which may explain why we found a lower TE content on the X′ compared with the homologous X region. Understanding the dynamics of TE accumulation in this peculiar system will require a more contiguous assembly of the X′ as well as examination of other X′ chromosomes in Sciaridae species (see below).

### A Role for Adaptive Stepwise Expansion of the X′

Over the last decade, there has been a growing recognition of the importance of clusters of linked loci within inversion-based supergenes in driving the evolution of diverse and complex phenotypes. These include Batesian mimetic morphs of butterflies (Joron et al. 2011), divergent social behaviors in ants (Yan et al. 2020), mating compatibility in fungi (Branco et al. 2018), as well as several polymorphisms in birds including plumage color (Funk et al. 2021), reproductive strategies (Küpper et al. 2016), and sperm morphology (Kim et al. 2017). This study of a recent transition in the sex-determining system in flies presents another case. It has been argued that supergenes may be more widespread than previously recognized, that they are important for cosegregation of adaptive variation within a species, and that they may even occasionally result in the spread of complex phenotypes across species boundaries (Schwander et al. 2014; Thompson and Jiggins 2014).

The evolutionary trajectories of supergenes and sex chromosomes show similarities: Some supergenes have evolved in a stepwise manner or have undergone functional degradation, and sex chromosomes also play an important role in adaptation and speciation (Presgraves 2008; Tuttle et al. 2016; Branco et al. 2018; Kim et al. 2022). Furthermore, the evolutionary fates of inversions differ depending on whether they arise on sex chromosomes or on autosomes, with the probability of spread of an inversion through a population being higher on sex chromosomes. This is further affected by sex-biased migration patterns, dominance of locally adapted alleles, and chromosome-specific deleterious mutation load (Connallon et al. 2018). Indeed, X-linked genes are predicted to disproportionately contribute to local adaptation due to exposure of recessive alleles to selection, and sex-linked inversions are therefore more likely to sweep to fixation compared with autosomal inversions (Lasne et al. 2017).

For these reasons, X-linkage of this supergene in Sciaridae may have favored its initial emergence as well as its enlargement along the chromosome. Rather than one long paracentric inversion, our analysis suggests the X′ chromosome has undergone multiple rearrangements, which may be explained by multiple adjacent and/or overlapping inversions, smaller inversions nested within larger ones, or some combination thereof, which have accumulated in a stepwise process to suppress recombination along the chromosome. Some of the smaller strata we identified appeared to be far less diverged than others, which may represent uninverted gaps between inversion breakpoints. Alternatively, the inversion(s) of the X′ may lead to complex pairing with the X, which may result in varying recombination rates along the chromosome and produce regions of differing divergence. Further resolution of the X′ structure will be required to determine the precise formation of the chromosome. Nonetheless, it appears that the rearrangements on the X′ accumulated rapidly less than 0.5 Ma. Expansion of the nonrecombining region through additional inversions may have adaptively captured female-beneficial alleles at nearby loci, as sex chromosome evolution theory posits (Charlesworth and Charlesworth 1980; Rice 1987; Charlesworth et al. 2005), although this may be hindered by genetic conflict between XX and X′X females.

An alternative explanation for the stratification of the X′ is that sex determination relies on more than one locus, that is, it is polygenic, and that successive inversions have emerged to control the sex ratio. Among digenic Sciaridae, sex ratios vary significantly: In *Bradysia ocellaris* and *Bradysia matrogrossensis*, broods frequently depart from the expected 50:50 sex ratio and are often heavily skewed in either direction (Rocha and Perondini 2000). The sex ratio in *B. ocellaris* is heritable, and majority male production can evolve from majority female production and vice versa in as few as six generations (Davidheiser 1947). Taken together, these observations suggest that multiple loci are involved in sex determination. Monogenic Sciaridae presumably evolved from digenic ancestors, which may have occurred through the adaptive linkage of sex-determining alleles through inversions. The young age of the X′ indicates that repeated evolution of monogeny in Sciaridae may have been favored under certain circumstances over the ancestral digenic sex determination system.

### Evolutionary Perspectives on Monogenic Reproduction in Fungus Gnats

Within the fungus gnat clade Sciaridae, origins of monogeny and the relationship between the monogenic and digenic reproductive strategies remain poorly understood. At least one other monogenic species, *B. impatiens*, is known to harbor an X-linked inversion polymorphism (Carson 1946). Monogeny also occurs in many other Sciaridae, including other *Bradysia* species, but also in more distantly related members of other genera such as *Lycoriella* and *Corynoptera* (Metz 1938). In this respect, our finding that the *B. coprophila* X′ chromosome evolved <0.5 Ma has intriguing consequences for understanding the evolution of this reproductive strategy. Unlike the *B. coprophila* X′, the *B. impatiens* X′ nonrecombining region is terminal (Carson 1946), suggesting that the X′ chromosomes in the two species may not be homologous by descent (alternatively, the region has expanded in *B. impatiens* or the terminal portion has reinverted in *B. coprophila*). Another possibility is that the X′ chromosome in *B. coprophila* may be older than our findings suggest but appears younger due to occasional recombination through gene conversion or double crossovers, which occur within large inversions (Navarro et al. 1997). While crossing-over requires synapsis between chromosomes, gene conversion, that is, the nonreciprocal copying of stretches of sequence between sister chromatids to repair mismatch errors during replication, does not (Szostak et al. 1983; McMahill et al. 2007). Furthermore, in *B. impatiens*, dicentric chromatids were observed to form through pairing between the X and X′ along the length of the inversion (Carson 1946). If such pairing occasionally occurs in *B. coprophila*, it may prevent sequence divergence between the two chromosomes.

Nonetheless, the distribution of monogenic reproduction among sciarids indicates multiple evolutionary origins. For example, within *Bradysia* alone, both monogenic and digenic species exist, and the same pattern is found within other genera (Metz 1938; Steffan 1974). If sex determination involves multiple loci, inversions may have emerged in some lineages to fix the production of sex-biased broods in a particular direction. However, this raises the question: What drives the turnover between reproductive strategies in this clade? Haig (1993) suggested that female production evolved as a response to a male-biased sex ratio. Fungus gnats carry a unique type of chromosome only found in the germline (germline-restricted chromosomes [GRCs]), in addition to their sex chromosomes and autosomes. The GRCs are disproportionately transmitted by males and so may have distorted the sex ratio in their favor. Presence of GRCs in Sciaridae does appear to correlate with monogenic reproduction, and many (but not all) digenic species lack them (Hodson and Ross 2021). In support of this is the observation that a monogenic lab-reared line of *B. impatiens* reverted to digenic reproduction following loss of its GRCs (Crouse et al. 1971). However, the function of the GRCs remains unknown. Interestingly, the GRCs of *B. coprophila* were recently found to have introgressed into Sciaridae following an ancient hybridization event with Cecidomyiidae (Hodson et al. 2022), a clade that shares many features with Sciaridae including paternal genome elimination, GRCs, sex determination by chromosome elimination, and monogenic reproduction (Benatti et al. 2010). It is thus tempting to speculate that GRCs may have spread throughout Sciaridae via similar introgression events and that this may have also facilitated the spread of monogenic reproduction.

Most or our knowledge about sex determination in Sciaridae comes from the study of several closely related *Bradysia* species (Metz 1938), though the more early diverging *Trichosia splendens* is also known to share the strange genetic features of *Bradysia* (Fuge 1994). These diverged sciarid genera have the same X-linked Muller elements (A and E), although *Phytosciara flavipes* has X-linked portions of other elements, which indicates there may be some different derived states (Anderson et al. 2022). It will thus be important to survey a wider sample of sciarid species to obtain a more comprehensive understanding of sex determination and sex chromosome evolution in this clade. Nonetheless, if female-determining inversions were to repeatedly evolve, individuals lacking inversions would be selected to increase their male production as an evolutionary response, with the expected result being that the X′X genotype is maintained at 50% in the population by frequency-dependent selection. However, the fact that we observe X′ degeneration could mean that X′X females will, over time, have reduced fitness, which should favor the invasion of individuals capable of digenic reproduction, unless occasional recombination keeps the X′ from degrading significantly or DC mechanisms are able to evolve. Future work on the role of GRCs in sciarid sex determination, and on the relationship between the unusual genetic aspects across different sciarid species, will be required to elucidate the origins and turnover of sex determinations strategies in this clade.

## Materials and Methods

### Data Collection

The *B.* (formerly *Sciara*) *coprophila* strain used in this study was obtained from the *Sciara* stock center at Brown University (https://sites.brown.edu/sciara/). Data were produced at Edinburgh University and the Carnegie Institution for Science in Baltimore. At Edinburgh, DNA was extracted using the Qiagen DNeasy Blood and Tissue Kit, modified for high-molecular weight (HMW) extractions. DNA from 50 to 60 X′X heads (i.e., soma) was used for sequencing on the Illumina NovaSeq S1 platform for paired-end 150-bp reads with 350-bp inserts; DNA from 30 to 40 X′X heads was used for PacBio Single-Molecular, Real-Time long-read sequencing. DNA samples were quantified using the Qubit (Thermo Fisher). HiC data were sequenced from 50 whole X′X females, which were ground using a DiagoCine Powermasher II with a Biomasher II attachment; libraries were prepared and sequenced by Science for Life Laboratory in Stockholm, Sweden. Illumina data from males (X0) previously generated for (Hodson et al. 2022) were used for the k-mer analysis (see below). At Carnegie, DNA was extracted using DNAzol (Thermo Fisher) from 20 to 36 pooled whole-body individuals, two replicates per genotype (X′X, XX, and X0), quantified with the Qubit, analyzed for purity with Nanodrop (Thermo Fisher), analyzed for HMW integrity with 0.5% agarose gel electrophoresis, prepared for sequencing using Illumina Nextera reagents, and sequenced on the Illumina NextSeq platform to generate 75-bp paired-end reads of ∼150- to 400-bp fragments. X0 PacBio data were from male embryos and were generated previously as part of assembling the original somatic reference genome (Urban et al. 2021). X0 HiC data were from male pupae and were generated previously for chromosome-scale scaffolding of the somatic reference genome (Urban et al. 2022). Illumina and HiC reads were adapter and quality trimmed using fastp v0.2.1 (Chen et al. 2018), and quality was assessed before and after trimming using FastQC (Andrews 2010).

### Analysis of X-X′ Divergence and Evolutionary Strata

To identify SVs, 75-bp paired-end Illumina reads and PacBio reads from X′X and X0 samples were aligned to the X0 reference genome (Urban et al. 2022) with BWA-MEM (Li 2013) to force-map X′ reads to the X chromosome. SAMtools (Li et al. 2009) was used to sort, merge, and index BAM files prior to calling SVs from Illumina alignments with Smoove v0.2.8 (Layer et al. 2014; Pedersen et al. 2020) and PacBio alignments with Sniffles v2.0 (Smolka et al. 2022). svtools (Larson et al. 2019) was used to convert variant files to bedpe files. To target fixed variants between X and X′, at least four reads were required to support a variant call. The R/Bioconductor (R Core Team 2022) package Sushi (Phanstiel et al. 2014) was used to plot SVs. HiC reads were aligned to the reference genome using Juicer (Durand et al. 2016), and HiC contact heatmaps were produced using the script HiC_view.py (Mackintosh et al. 2022). To call SNVs, the Illumina alignments were processed with Picardtools (Anon 2019) before calling variants with the GATK-4 best practices pipeline (McKenna et al. 2010; DePristo et al. 2011). The Python scripts parseVCF.py and popgenWindows.py (https://github.com/simonhmartin/genomics_general) were then used to parse the variant files and calculate the density of SNVs (i.e., heterozygous sites) across 100-kb windows, respectively. The R (R Core Team 2022) change-point package bcp (Erdman and Emerson 2008) was used to identify breakpoints between putative evolutionary strata.

Two methods were employed to estimate the ages of strata. We assumed a neutral mutation rate and a similar mutation rate to *Drosophila melanogaster*, that is, between 2.8 × 10^−9^ (Keightley et al. 2014) and 4.9 × 10^−9^ (Assaf et al. 2017), and a 24- to 40-day generation time for *B. coprophila* (supplementary Text S5, Supplementary Material online). First, the density of heterozygous sites (i.e., number of heterozygous sites divided by the number of homozygous and heterozygous sites) across 100-kb windows in each stratum (see above) was taken as a proxy for *Dxy*. Generations since divergence were calculated as “*Dxy*/2 ∗ *r*,” where *r* is mutation rate. Second, we targeted only synonymous (neutral) mutations. SnpEff (Cingolani, Patel, et al. 2012) and SnpSift (Cingolani, Platts, et al. 2012) were used to annotate variants and count synonymous variants per gene. The script partitionCDS.py (Mackintosh et al. 2022) was used to annotate degeneracy for all genic sites. Divergence in generations for single-copy homologs within each stratum was then calculated as “*V*/(2 ∗ *r* ∗ *S*),” where *V* is the number of synonymous variants, *r* is mutation rate, and *S* is the number of synonymous sites.

### Genome Assembly and Annotation

A genome assembly was initially generated de novo from PacBio reads from X′X females, but only ∼3.6 Mb of sequence from this assembly could be assigned to the X′ inversion because high sequence similarity between X and X′ chromosomes and high read error rates (∼11–16% on average) caused them to collapse upon assembly (supplementary Text S2 and fig. S1, Supplementary Material online). To assemble the X′ inversion, putative X′-specific 27-mers were identified using KAT (Mapleson et al. 2016), counted using KMC3 (Kokot et al. 2017) and FASTA files of 27-mers were obtained using custom Python scripts (Hodson et al. 2022). Cookiecutter (Starostina et al. 2015) was used to bin X′ and non-X′ (A + X) reads from X′X Illumina reads, whereby reads are binned if they contain k-mers from a given k-mer library. The X′-specific reads were assembled using SPAdes (Bankevich et al. 2012) to yield 61.77 Mb spanning 42,564 contigs with an N50 of 10.32 kb and a largest contig length of 86.95 kb. These contigs were then stitched together with the reference-based scaffolder RagTag (Alonge et al. 2021) using the regular X chromosome as a reference (Urban et al. 2022) into a single 52.43-Mb scaffold containing 9,409 gaps. The X′ scaffold was then combined with the male X0 genome to produce an X′X genome. PacBio long reads from an X′X female were mapped to the X′X genome with minimap2, and gaps were plugged using Racon (Vaser et al. 2017). Illumina reads from an X′X female were subsequently mapped to the X′X genome with minimap2 (Li 2018), and Racon (Vaser et al. 2017) was used to polish the assembly. After gap-plugging and polishing, the X′ inversion scaffold totaled 54.75 Mb and contained 3,486 gaps.

The genome was soft-masked prior to annotation. To this end, de novo repeat families were created using RepeatModeler v2.0 (Flynn et al. 2020), which were combined with known dipteran repeat families from RepBase (Bao et al. 2015). RepeatMasker v4.0 (Smit et al. 2015) was used to soft-mask the genome, which was then annotated using BRAKER2 (Stanke et al. 2008; Lomsadze et al. 2014; Hoff et al. 2016; 2019; Brůna et al. 2021) with RNAseq alignments and homology-based data sets (supplementary Text S6, Supplementary Material online). Functional information for the 26,887 protein sequences in the resulting BRAKER2 gene annotation set was then obtained by finding the best BlastP (Altschul et al. 1990) hits in several protein databases and with InterProScan (Jones et al. 2014, supplementary Text S5, Supplementary Material online). The reasonaTE module of the TranposonUltimate v1.03 pipeline (Riehl et al. 2022) was used to annotate TEs in the genome (supplementary Text S7, Supplementary Material online).

### Analysis of Functional Degradation

Homologous X-X′ gene copies were identified with OrthoFinder (Emms and Kelly 2019). To identify disrupted X′-linked genes, genotyped variant files for X′X and X0 75-bp paired-end Illumina reads mapped against the Bcop_v2 (X, II, III, and IV) genome (as described above) were filtered for SNVs and indels using SnpEff (Cingolani, Patel, et al. 2012) and SnpSift (Cingolani, Platts, et al. 2012). SNV and indel counts for 2,321 single-copy homologs were then analyzed in RStudio (R Core Team 2022). Two replicates of each genotype (X′X and X0) were aligned, and only consensus variant calls between replicates were considered to identify fixed differences between X and X′ and exclude low-frequency variants. Variants specific to the X′ were identified by excluding common variants also found with X0 male data, which may represent polymorphisms on the X or errors in the X chromosome reference sequence (Urban et al. 2022). Genes with loss or gain of start or stop codons or indels causing frameshifts were counted as disrupted.

To identify silenced genes, RNAseq reads were binned as X or X′ in origin using elements of a pipeline developed for (Marshall et al. 2020) to avoid mismapping between the two chromosomes (supplementary Text S8, Supplementary Material online). Expression of the 2,321 single-copy homologs was quantified using Kallisto (Bray et al. 2016), and counts were normalized with EdgeR (Robinson et al. 2010). Genes with zero counts of transcripts per million (TPM) and those with TPMs in the bottom 0.1% of nonzero TPM counts within a sample (to account for stochastic mismapping of RNAseq reads) were assumed to be nonexpressed. X′ genes were counted as “silenced” if the X copy was expressed but the X′ copy was not, and if genes were both silenced and contained pseudogenizing mutations, we counted them as “silenced and disrupted.” Analysis and plotting of counts was carried out with R Studio (R Core Team 2022).

To examine DC of X genes that have corresponding degraded X′ homologs, the same pipeline (supplementary Text S8, Supplementary Material online) was used to compare expression of X-linked genes between X′X and XX females (supplementary Text S9, Supplementary Material online). In X′X females, X-linked genes with degraded X′ homologs should have upregulated expression if they are dosage compensated to match expression in XX females. Upregulated genes were identified as those with a log2fold-change (FC) of >0.5.

## Supplementary Material

msad148_Supplementary_Data

## Data Availability

Raw Illumina and PacBio data used in this project have been submitted to NCBI BioProject and SRA databases under accession PRJNA953429. The X′ scaffold has been deposited at DDBJ/ENA/GenBank under accession JASKUS000000000. The version described in this paper is version JASKUS010000000. Code used in analyses is available in a public GitHub repository (https://github.com/RossLab/Xprime_paper).
