# Supplementary material for: Recent Evolution of a Maternally Acting Sex-Determining Supergene in a Fly with Single-Sex Broods
Source: Mol Biol Evol. 2023 Jun 23;40(7):msad148. doi: 10.1093/molbev/msad148 (PMC10321493; doi:10.1093/molbev/msad148)
Supplement: msad148_Supplementary_Data [file msad148_supplementary_data.docx]

**Supplementary information for:**

Recent evolution of a maternally-acting sex-determining supergene in a fly with single-sex broods

Robert B. Baird^1^*, John M. Urban^2^*, Andrew J. Mongue^1^, Kamil S. Jaron^3^, Christina N. Hodson^4^, Malte Grewoldt^5^, Simon H. Martin^1^ and Laura Ross^1^.

1. Institute of Evolutionary Biology, University of Edinburgh, Edinburgh, EH9 3JT, UK

2. Department of Embryology, Carnegie Institution for Science, Howard Hughes Medical Institute Research Laboratories, Baltimore, Maryland 21218. United States of America

3. Tree of Life Programme, Wellcome Sanger Institute, Wellcome Genome Campus, Hinxton, Cambridge CB10 1SA, UK

4. Department of Zoology, University of British Columbia, Vancouver, V6T 1Z4, Canada

5. Department of Molecular Biology and Genetics, Aarhus University, 8000 Aarhus, Denmark

*These authors contributed equally to this work.

Corresponding author: Robert B. Baird

**Email:**  robert.baird@ed.ac.uk

This PDF file includes:

Supplementary text 1-9

Supplementary figures 1-5

Supplementary tables 1-7

**Supplementary text 1. Mapping rates of X0, XX and X’X libraries.**

In the *B. coprophila* system, males are X0 and females are either XX or X’X; the X and X’ are distinguished by a large non-recombining region. X0 and XX individuals have essentially the same genome. Therefore, when mapping DNAseq libraries of each genotype to the reference genome, which is assembled from male data and so has the X but not the X’ (Urban et al*.*, 2021; 2022), mapping rates are expected to be the same between X0 and XX libraries. X’X libraries should have a slightly lower mapping rate due to some expected level of divergence between the X and X’ chromosomes. However, while we found lower mapping rates for the two X’X libraries (95.64% and 95.96%) compared to the XX libraries (96.76% and 96.61%), we unexpectedly found that X0 libraries had the lowest mapping rates (93.23% and 93.85%). This ~3% difference in mapping rate, consistent across replicates, warrants an explanation.

To resolve this question, we focused on two potential explanations for the observed mapping rates. Firstly, the differences may be due to human and/or microbial contamination or differences in microbiome content between the sexes. Secondly, the male libraries may contain a higher proportion of reads that originate from the germline-restricted chromosomes (GRCs, Hodson et al., 2022). Though both sexes possess GRCs, and although the germline likely constitutes a small proportion of total cells, males have many more sperm cells than females do egg cells. Furthermore, sperm cells have two copies of the GRCs while eggs only have one (Goday and Esteban 2000). Since the GRCs are not included in the *Bradysia coprophila* reference genome, we reasoned that some of the difference may be due to a higher GRC content in the male libraries.

To assess the extent of contamination, we analyzed the unmapped reads from each library. Unmapped read were extracted from BWA-mapped (Li 2013) alignment files using SAMtools (Li et al. 2009). Kraken2 (v 2.1.2, Wood et al. 2019) was used to analyze read pairs and singletons separately using the PlusPF 20210517 database (<https://genome-idx.s3.amazonaws.com/kraken/k2_pluspf_20210517.tar.gz>). Sensitive and lightweight parameters were used (-confidence 0 –minimum-hit-groups 1 –memory-mapping). Classified and unclassified reads were written to FASTQ files (--classified-out / --unclassified-out). The Pavian R package (Breitwiese and Salzberg 2016) was used to interpret and plot the Kraken2 results. The contamination report showed that the X0 libraries had a higher proportion of contaminating chordate (likely human) and bacterial DNA compared to the XX and X’X libraries. Moreover, approximately 2.93% of read pairs from the X0 library were identified as contaminants (of which the vast majority was bacterial), compared to 1.30% and 1.44% for the XX and X’X libraries, respectively (**Table S7**).

In an attempt to classify some of the remaining unmapped reads, 1000 unmapped reads that were not classified by Kraken2 were randomly sampled from each mate in unmapped-unclassified pairs separately and independently. Each random sample of unmapped-unclassified reads was subject to mapping with BLAST (v 2.13.0+, Altschul et al. 1990) to the full NT dataset (downloaded/updated 2023-03-29) using sensitive parameters for short sequences. The taxonomic identifier (taxid) from the top hit for each read was used to classify them, using custom Python scripts (<https://github.com/JohnUrban/sciara-project-tools/tree/master/taxon>) that depend on the ‘cogent’ Python library (Knight et al. 2007), specifically returning the node in the taxonomic tree closest to ‘*Bradysia coprophila*’. If the closest node was at least as close to *Bradysia* as Arthropoda (e.g. Insecta or Diptera), then the read was not considered a contaminant. Using this method and extrapolating to the rest of the input libraries, we classified a further 2.51% of X0 reads as contaminants, compared to 1.25% and 1.29% for the XX and X’X libraries, respectively (**Table S7**). Together, from Kraken2 and BLAST, we estimate that 89.6%, 87.3% and 85.5% of the X0, XX and X’X unmapped reads, respectively, were from contaminating sources, which translates to 5.4%, 2.5% and 2.7% of the corresponding input libraries. Thus, contamination explains ~92-94% of the difference in mapping rates between males and females.

We also mapped the remaining unmapped-unclassified reads to the GRC scaffolds (Hodson et al., 2022) using BWA-MEM (Li 2013) and obtained mapping rates using SAMtools (Li et al. 2009). We identified more GRC reads in the male libraries compared to the female libraries, though they only comprised a small proportion of total reads (0.30% for X0, 0.09% for XX and 0.12% for X’X). In total, we explained approximately 90-95% of the unmapped reads as contaminants or GRC in origin, with the vast majority being explained by bacterial contamination.

**Supplementary text 2. *De novo* assembly of reads from X’X individuals.**

Initially, PacBio sequel 3.0 reads from X’X females were assembled *de novo* using wtdbg2 (Ruan and Li 2020) with a lower read length threshold of 2500bp, into 2382 contigs spanning 307Mb with an N50 of 353Kb. The assembly was polished twice with the PacBio reads and three times with 150bp Illumina reads also from X’X individuals using Racon (Vaser et al. 2017). Blobtools v1.1.1 (Laetsch and Blaxter 2017) and BUSCO v4 with the insecta_odb10 dataset (Seppey et al. 2019) were used to assess assembly quality and a custom R script was used to remove contaminant and low-coverage contigs. The final BUSCO score was 96.9% (93.5% complete and single-copy BUSCOs, 3.4% complete and duplicated BUSCOs). Scaffolds were assigned to chromosomes based on differences in X0 male and X’X female read coverage. To this end, reads were mapped to the assembly using BWA-MEM (Li 2013) and per-based genome coverage was calculated using BEDTools (Quinlan and Hall 2010). A custom R (R Core Team 2022) script was used to assign scaffolds to chromosomes: scaffolds with a relative coverage of 2x in both males and X’X females were assigned as autosomal; scaffolds with a relative coverage of 1x in males and 1-2x in X’X females were assigned to the X chromosome; scaffolds with low (<0.5x) male coverage and 1x coverage in females were assigned to the inversion (**Figure S1**). A custom R script was used to calculate and plot mean coverage across 20Kb windows of the assembly. In total, only 3.63Mb of sequence was assigned as X’ inversion sequence using this method. The amount of sequence assigned to the autosomes and X chromosome was similar to that assigned in (Urban et al. 2021a).

**Supplementary text 3. Binning reads with K-mers.**

To assign K-mers as X’-specific, 27-mers were counted in 150bp Illumina read libraries for female-producing female (X’X) and male (X0, from 9) datasets using KMC (Kokot et al. 2017). 27-mers with a frequency of 0 in the X0 library and >5 in the X’X library (to exclude K-mers containing read errors) were assigned as X’-specific; this decision was based on the distribution of 27-mer frequencies visible in **Figure 4.** The cookiecutter (Starostina et al. 2015) option ‘extract’ was used to pull reads from the raw read file. Cookiecutter creates two separate files: one for all reads that contain a matching K-mer and one containing reads whose mate contained a matching K-mer; both files were combined into one because a read should originate from the same chromosome as its mate. This was done for both 150bp and 75bp Illumina libraries that were available. Extracting putative X’ reads with the X’-specific 27-mer library resulted in around 10-12% of reads being pulled; this was roughly in line with the expected size of the X’ relative to the rest of the haploid genome (around 40-60Mb in the expected 330-350Mb X’X genome, or 12-17%). When attempting the same process for PacBio sequel 3.0 error-prone reads, significantly more reads were pulled than expected: around 23% of X’-specific reads. We also identified putative autosomal- and X-specific K-mers based on the frequencies visible in **Figure 4**, similar to the methods used in (Hodson et al. 2022), and used these to extract putative autosomal and X reads. For Illumina reads the results were broadly as expected: around 60% for autosomal reads and 13% for X reads. Longer K-mer lengths resulted in very few reads being extracted. It is likely that shorter K-mers occur more often by chance in long reads and result in undesired reads being pulled, while longer K-mers are unlikely to have any matches due to the high error rate of the PacBio reads. For this reason, we decided to competitively map the PacBio reads post-assembly and use them for plugging gaps (**Figure S4**).

**Supplementary text 4. Identification of degraded genes of interest.**

To identify degraded genes that may be of interest, we searched among functional annotations of genes that we classified as degraded using keywords. To identify genes potentially involved in sex determination by chromosome elimination, the keywords used were: ‘chromosome’, ‘chromatid’, ‘chromatin’, ‘cohesion’, ‘condensation’, ‘segregation’, ‘centromere’, ‘centrosome’ and ‘spindle’. To identify candidates responsible for the wavy wing mutation, we searched for the keyword ‘wing’. To identify potential genes with roles in male fertility, the keywords used were ‘sex’, ‘sperm’, ‘fertility’, and ‘meiosis’.

**Supplementary text 5. Fly husbandry.**

*Bradysia* (formerly Sciara) *coprophila* flies have been reared in laboratory conditions since the 1920s (Metz 1925). Colonies can be successfully maintained in a variety of ways but are healthiest when kept at around 18-21°C and around 70% relative humidity, and when conditions are not too crowded. At Edinburgh University, colonies are maintained using matings between a single female and two males (to protect against sterility) and are reared on biological agarose in 25mm x 95mm glass vials at 18°C. At the Carnegie Institution for Science in Baltimore, colonies are maintained using mass matings controlled for the sex of offspring (5 males plus 5 female-producing or 5 male-producing females) and are reared in 60z Square Bottom Polypropylene Bottles (Genesee Scientific) capped with Flystuff Flugs (Genesee Scientific) at 21°C and >70% relative humidity. At both institutions, flies are kept on 2.2% Bactoagar and are fed a mixture of yeast, powered mushroom, powdered spinach or nettle and ground straw while larvae.

Hungerford (Hungerford 1916) first reported the generation time of *B. coprophila* (from fertilized egg to eclosion) at between 24 and 32 days. Rieffel and Crouse (Rieffel and Crouse 1966) timed developmental stages so as to reflect the minimum length of each stage in ideal conditions, i.e. when crowding, food scarcity, competition and temperature do not interfere with normal development. These minimum developmental stages summed together are 24.6 days for males and 26.6 days for females. Having raised generations of *B. coprophila* in the lab for several years, the authors of this paper have observed the minimum generation time as approximately 28 days, though adults from a single progeny often continue to eclose for up to a week. The generation time fluctuates with temperature: the authors have previously raised *B. coprophila* at 25°C, which speeds up development by at least a few days, and it has previously been noted that rearing *B. coprphila* and *B. ocellaris* at temperatures as low as 12°C is possible but significantly delays development (Smith-Stocking 1936; Nigro et al. 2007). *B. coprophila* have also been observed to survive up to several months when refrigerated (R. B. Baird, C. N. Hodson & L. Ross, unpublished observations; 8). This potential adaptation to cold temperatures may suggest that larvae lie dormant for long periods in winter, which might increase the average generation time. Harsher conditions such as crowding and food scarcity do also appear to extend the generation time beyond 40 days (J. M. Urban & R. B. Baird, unpublished observations). On the other hand, the generation time may be shorter in regions with warmer climates. Observations of other, closely-related species report varying generation times: 26-48 days or *B. odoriphaga* (Li et al. 2015), 14 days for *B. impatiens* (Kennedy 1973), 25 days at 20-25°C or 3-4 weeks at 22-24°C for *B. paupera* (Mansilla et al. 2001) and 26-28 days at 25°C for *B. difformis* (Villanueva-Sánchez et al. 2013); see (Katumanyane et al. 2018 for a review). Overall, it is difficult to estimate the generation time that best approximates *B. coprophila* development in nature, but in light of the literature cited here and observations made by the authors we decided to use a generation time 24-40 days to calculate divergence between the X and X’ chromosomes in *B. coprophila* in years.

**Supplementary text 6. Genome annotation.**

The RNAseq data used in annotation of the genome (including autosomes II, III, IV, X and the X´ sequence) was obtained from publicly available data previously produced by Urban *et al.* (2015; 2021a; Urban 2021b) and unpublished datasets produced by the Ross Lab. This included 18 RNAseq datasets from four life stages (3 x male and 3 x female embryos, 2 x male and 2 x female larval, 2 x male and 2 x female pupal, 2 x male and 2 x female adult, (Urban et al. 2021b), 15 datasets from dissected larval salivary glands (pooled individuals, (Urban et al. 2015), 6 datasets from radiated larvae (pooled female individuals, (Urban et al. 2021a), 6 datasets from male and female somatic tissue (3 x male, 3 x female larval/early pupal), as well as 12 datasets from early (0-8h) embryos (6 x male, 6 x female, unpublished data). All RNAseq datasets were aligned to the genome using STAR (Dobin et al. 2013) prior to being fed to BRAKER2. Homology-based datasets included all OrthoDB v10 Diptera protein sequences (751660 sequences,(Kriventseva et al. 2019), all Uniprot Diptera protein sequences (7053 sequences, (Uniprot Consortium 2015) and all Refseq Diptera protein sequences including all non-canonical isoforms (1575334 sequences, (Pruitt et al. 2007).

Functional information for the 26887 protein sequences in the BRAKER2 gene annotation set was obtained by finding the best BLASTP (Altschul et al. 1990) hits in several protein databases and with InterProScan. Specifically, all BLASTP hits (-word_size 3 -evalue 1e-2) for all 26887 proteins were found in (i) the *Drosophila melanogaster* r6.45 (dos Santos et al. 2014) proteome, (ii) Non-redundant UniProtKB/SwissProt (Uniprot Consortium 2020) protein sequences (ftp://ftp.ncbi.nlm.nih.gov/blast/db/swissprot.tar.gz),  (iii) the NCBI Landmark database for SmartBLAST (ftp://ftp.ncbi.nlm.nih.gov/blast/db/landmark.tar.gz), (iv) the NCBI RefSeq (O’Leary et al. 2015) protein database (ftp://ftp.ncbi.nlm.nih.gov/blast/db/refseq_protein.*.tar.gz), (v) the NCBI Non-Redundant (nr) protein database (ftp://ftp.ncbi.nlm.nih.gov/blast/db/nr.*.tar.gz), (vi) OrthoDB v10 (Kriventseva et al. 2019), (vii) and gene annotations from the original *Bradysia coprophila* reference genome (40). The best BLASTP hit for all 26887 proteins in each database was found by taking the hit with the highest bitscore. Hits with bitscores lower than 50 or e-values higher than 0.0005 were removed from consideration. The 26887 protein sequences were also extensively analyzed using the InterPro (Blum et al. 2020) protein family and domain database and InterProScan version 5.56-89.0 (-dp -iprlookup -goterms -f tsv,xml,json,gff3, (Jones et al. 2014) allowing all analyses to be run: CDD, Coils, Gene3D, Hamap, MobiDBLite, PANTHER, Pfam, Phobius, PIRSF, PIRSR, PRINTS, ProSitePatterns, ProSiteProfiles, SFLD, SignalP, SMART, SUPERFAMILY, TIGRFAM, TMHMM. Software licenses were obtained where relevant (SignalP, Phobius, TMHMM).

**Supplementary text 7. Transposable element annotation.**

To annotate transposable elements (TEs) in the genome, the module reasonaTE of the TranposonUltimate v1.03 pipeline (Riehl et al. 2022) was used (**S7 Text**). Combining the different annotation approaches and sensitivities of RepeatMasker (v4.1.2, (Smit et al. 2015), RepeatModeler (v2.0.2a, (Flynn et al. 2020), LTRharvest (2.9.0, (Ellinghaus et al. 2008), TIRvish (v.1.10.5, (Gremme et al. 2013), SINE-Scan (v1.1.2, (Mao and Wang 2017), HelitronScanner (v1.0.0, (Xiong et al. 2014), MiteFinderII (v1.0.0, (Hu et al. 2018), MITE-Tracker (v1.0.1, (Crescente et al. 2018), TransposonPSI (v1.0.0, https://transposonpsi.sourceforge.net/) and NCBICDD1000 (v1.0.1, (Lu et al. 2020) it enables in-depth analysis of various classes of TEs in the genome. R Studio (R Core Team 2022) was used to plot TE distribution across chromosomes.

**Supplementary text 8. Binning RNAseq reads to identify silenced genes and examine dosage compensation.**

To identify silenced genes, we parsed X and X´ RNAseq reads using elements of a pipeline from (Marshall et al. 2020, https://github.com/MooHoll/Parent_of_Origin_Expression_Bumblebee) to avoid mismapping between the two chromosomes. To this end, we created an N-masked version of the X chromosome to mask SNVs between the X and X´ by mapping reads from X´X individuals to the X0 reference genome (Urban et al. 2021b) using Bowtie2 (Langmead and Salzberg 2012), processed BAM files using Picardtools (Anon 2019) and SAMtools (Li et al. 2009) and called and filtered SNVs using freebayes (Garrison and Marth 2012) and VCFtools (Danecek et al. 2011) respectively. BEDtools (Quinlan and Hall 2010) was used to identify X- and X´-specific variants and to N-mask those variants on the X chromosome prior to mapping RNAseq reads. We mapped RNAseq reads from pooled female data from four life stages: embryo, larva, pupa and adult (Urban et al. 2021b) to the N-masked genome using STAR (Dobin et al. 2013) and subsequently assigned RNAseq reads to the X or X´ chromosome using SNPsplit (Krueger and Andrews 2016). Gene expression was quantified using Kallisto (Bray et al. 2016) with the 2321 singlecopy homologs we identified (see main text) as a Kallisto index, and counts were normalized using EdgeR (Robinson et al. 2010). Genes with zero counts of TPM (transcripts per million) were assumed to be non-expressed. We also included genes with TPMs in the bottom 0.1% of non-zero TPM counts within a sample as non-expressed to account for stochastic mismapping of RNAseq reads. Analysis and plotting of counts was carried out with R Studio (R Core Team 2022) with use of the ggplot2 package (Wickham 2016).

To examine dosage compensation of X-linked genes with degraded X´-linked homologous in the X´X females, we applied the same pipeline to extract RNAseq reads originating from the X chromosome in those females. Kallisto (Bray et al. 2016) was used to quantify the resulting X reads against the 2321 X-linked single-copy homologs and normalization between samples was carried out with EdgeR (Robinson et al. 2010). We subsequently compared expression of X-linked genes in X´X versus XX females, with the expectation that X-linked genes with degraded X´ homologs should be upregulated in X´X females if they are dosage compensated.

**Supplementary text 9. Data used to examine dosage compensation.**

To investigate potential dosage compensation (DC) of X-linked genes with degraded X´ homologs, we used RNAseq data from early (0-8h) embryos. This dataset was generated by R. B. Baird for the purpose of another, unpublished study. Adult females were mated with males (generally ~10 females with ~3 males in a tube), and after ~18-24 hours, females were pinned to a petri dish containing 2.2% Bactoagar and egg laying was induced by crushing the head slightly. An entire clutch (50-100 eggs) is usually laid within the next 2 hours. Three replicates from two time stages were collected: 0-4h and 4-8h after egg deposition (AED), for the eggs of each female genotype (X´X and XX), resulting in 12 samples in total. For each sample, around ~600-800 eggs pooled from ~15 individuals were collected and sequenced for 15Gb (50m reads) of paired-end 150bp RNAseq reads on the Illumina Novaseq S1 platform.

We used these data to examine expression of X-linked genes in X´X versus XX females because data for female adults (or other developmental stages) separated by genotype are not yet available, and these early embryos are likely to contain exclusively maternal transcripts. In *Drosophila*, zygotic genome activation (ZGA) occurs in two waves at mitotic cycle 8 (60 genes) and following cellularization at mitotic cycle 14 (over 1000 genes, 22). De Saint Phalle & Sullivan (de Saint Phalle and Sullivan 1996) report that cellularization in *B. coprophila* occurs during interphase of nuclear cycle 11, which begins at 9.3 +/- 1.1 hours AED. Cell cycle 12 begins at 11.4 +/- 1.4 hours AED. However, as in *Drosophila*, de Saint Phalle & Sullivan (de Saint Phalle and Sullivan 1996) report that the germline nuclei cellularize slightly earlier, at cell cycle 7, which occurs around 4.2 +/- 0.5 hours AED. Nonetheless, if we assume that, as in *Drosophila*, ZGA occurs approximately when cellularization occurs, ZGA should begin for the majority of somatic genes by 12 hours AED.

Upon splitting RNAseq reads from the eggs of X’X mothers into X´ and X reads (see methods in **S6 Text**), similar proportions of reads were assigned to the X´ (5.67%) and X (6.17%) aside from one outlier sample (eggs of X´X females, 4-8h, replicate 2), which had 7.97% of reads assigned to the X and 3.61% assigned to the X´). This sample was excluded from further analysis. If transcripts were zygotic rather than maternal, then the number of X reads assigned should be three-fold higher for the X, because in the pooled eggs, maternal transcripts from X´X mothers will originate from the X´ and X in equal proportions. However, following ZGA, three times as many X transcripts should be produced for every X´ transcript, owing to half the eggs being XX and the other half X´X.

**Supplementary text 10. Removing adapter sequences found in the X’ scaffold.**

Upon submission of the X’ inversion scaffold to NCBI, five sequences were flagged as potential adapter contamination. All five sequences were shorter than 48 bp, at contig ends immediately adjacent to a gap, and were at the following locations within the scaffold: 8,259,796-8,259,842; 12,677,415-12,677,459; 30,859,720-30,859,766; 44,216,421-44,216,468; 53,963,934-53,963,981. These sequences may were present in the assembly prior to scaffolding/gap-filling/polishing, so may have been missed by the trimming software. We masked out these short sequences by extending the adjacent gaps to cover them as well.

**Supplementary table 1.** Assembly statistics and chromosome anchorage for the *B. coprophila* genome assembled *de novo* from PacBio long reads from X’X individuals.

| **Partition** | **Length (Mb)** | **N scaffolds** | **N50 (Kb)** | **Largest scaffold (Kb)** |
| --- | --- | --- | --- | --- |
| Autosomes | 215.15 | 1123 | 423.42 | 2141.89 |
| X chromosome | 72.84 | 864 | 205.13 | 995.08 |
| Inversion | 3.63 | 259 | 17.69 | 73.99 |
| Total | 291.63 | 2246 | 342.36 |  |

| **Supplementary table 2.** Data used to calculate age estimates for each stratum, and upper and lower estimates for neutral estimates (based on different estimates of mutation rate and generation time). Note that the numbers of synonymous sites are not integers because the weight of each site is normalized in accordance with codon degeneracy (see methods). | | | | | | | | | | |
| --- | --- | --- | --- | --- | --- | --- | --- | --- | --- | --- |
| **Stratum** | **Start (bp)** | **End (bp)** | ***Dxy* (het density)** | **N single-copy homologs within boundaries** | **N syn sites** | **N syn variants** | **Lower *Dxy* estimate (mya)** | **Upper *Dxy* estimate (mya)** | **Lower neutral sites estimate (mya)** | **Upper neutral sites estimate (mya)** |
| S1 | 4050001 | 13050000 | 0.0075 | 326 | 155620 | 1533 | 0.050 | 0.147 | 0.075 | 0.219 |
| S2 | 13050001 | 13550000 | 0.0040 | 15 | 11848.98 | 60 | 0.027 | 0.078 | 0.05 | 0.145 |
| S3 | 13550001 | 37350000 | 0.0123 | 904 | 460851.1 | 6903 | 0.083 | 0.241 | 0.113 | 0.329 |
| S4 | 37350001 | 38350000 | 0.0039 | 35 | 17063.14 | 95 | 0.026 | 0.077 | 0.034 | 0.099 |
| S5 | 38350001 | 40000000 | 0.0151 | 55 | 19150.58 | 420 | 0.101 | 0.295 | 0.149 | 0.434 |
| S6 | 40000001 | 40100000 | 0.0074 | 2 | 1162.64 | 3 | 0.049 | 0.144 | 0.05 | 0.147 |
| S7 | 40100001 | 49500000 | 0.0145 | 350 | 158696.8 | 3204 | 0.097 | 0.284 | 0.148 | 0.432 |
| S8 | 49500001 | 49600000 | 0.0006 | 0 | 0 | 0 | 0.004 | 0.011 | NA | NA |
| S9 | 49600001 | 52150000 | 0.0153 | 62 | 30364.9 | 692 | 0.102 | 0.298 | 0.162 | 0.474 |
| S10 | 52150001 | 52550000 | 0.0122 | 22 | 7521.66 | 111 | 0.082 | 0.239 | 0.119 | 0.346 |
| S11 | 52550001 | 54050000 | 0.0145 | 50 | 26014.18 | 427 | 0.097 | 0.284 | 0.141 | 0.412 |
| S12 | 54050001 | 55950000 | 0.0159 | 96 | 39556.22 | 873 | 0.107 | 0.312 | 0.171 | 0.499 |
| S13 | 55950001 | 57250000 | 0.0136 | 90 | 44562.88 | 830 | 0.091 | 0.266 | 0.132 | 0.386 |
| S14 | 57250001 | 58450000 | 0.0104 | 36 | 14329.12 | 236 | 0.070 | 0.204 | 0.146 | 0.425 |
| S15 | 58450001 | 61950000 | 0.0096 | 97 | 38924.46 | 429 | 0.065 | 0.188 | 0.075 | 0.218 |
| S16 | 61950001 | 62900000 | 0.0093 | 54 | 22126.82 | 346 | 0.062 | 0.182 | 0.102 | 0.297 |

**Supplementary table 3.** Number of genes within the X’ supergene sequence with each type of predicted pseudogenizing mutation.

| **Mutation type** | **N genes** |
| --- | --- |
| Stop codon gained | 37 |
| Stop codon lost | 10 |
| Start codon lost | 10 |
| Frameshift | 113 |
| Frameshift and stop codon gained | 15 |
| Frameshift and stop codon lost | 6 |
| Frameshift and start codon lost | 8 |
| Frameshift and stop codon gained and stop codon lost | 2 |
| Frameshift and stop codon gained and start codon lost | 1 |

**Supplementary table 4.** Functions of pseudogenized genes of interest.

| **Gene ID** | **Functionality** | **Annotation(s)** |
| --- | --- | --- |
| jg4815 | nonfunctional_disrupted | Similar to how Protein held out wings |
| jg6281 | nonfunctional_silenced | Similar to su(Hw) Protein suppressor of hairy wing |
| jg5727 | nonfunctional_disrupted | Similar to su(Hw) Protein suppressor of hairy wing |
| jg7913 | nonfunctional_disrupted | Similar to su(Hw) Protein suppressor of hairy wing |
| jg7400 | nonfunctional_disrupted | Similar to stan Protocadherin-like wing polarity protein |
| jg6904 | nonfunctional_disrupted | Similar to Etl1 SWI/SNF-related matrix-associated actin-dependent regulator of chromatin subfamily A containing DEAD/H box 1 homolog; Similar to Marcal1 SWI/SNF-related matrix-associated actin-dependent regulator of chromatin subfamily A-like protein 1 |
| jg6344 | nonfunctional_disrupted | Similar to Etl1 SWI/SNF-related matrix-associated actin-dependent regulator of chromatin subfamily A containing DEAD/H box 1 homolog; Similar to Marcal1 SWI/SNF-related matrix-associated actin-dependent regulator of chromatin subfamily A-like protein 1 |
| jg7872 | nonfunctional_silenced | Similar to dsx Protein doublesex |
| jg6244 | nonfunctional_disrupted | Similar to Crocc Rootletin; Ciliary rootlet component, centrosome cohesion |
| jg6043 | nonfunctional_silenced | Similar to TRAF3IP1 TRAF3-interacting protein 1; Microtubule-binding protein MIP-T3 C-terminal region |
| jg5804 | nonfunctional_disrupted | Similar to ncd Protein claret segregational |

**Supplementary table 5.** Upregulated X-linked genes with single-copy X’-linked homologs in X’X females.

| **Gene ID** | **Annotation(s)** |
| --- | --- |
| jg4857 | Similar to PARP3 Protein mono-ADP-ribosyltransferase PARP3 |
| Jg5988 | Similar to srfbp1 Serum response factor-binding protein 1;  Similar to F52C9.6 Putative uncharacterized transposon-derived protein F52C9.6 |
| Jg6324 | Protein of unknown function |
| Jg7315 | Similar to eff Ubiquitin-conjugating enzyme E2-17 kDa |

**Supplementary Table 6.** SV signal breakpoints associated with annotated repeats.
75% of the SV signals from discordant paired-end short read alignments had one or more breakpoints inside annotated repeats (compared to 59% at random), suggesting repeats (e.g. transposon sequences) are differentially distributed across the X’ compared to the X. These discordant paired-end mappings are not simply a problem of multi-mapping reads since (i) the Smoove pipeline filtered out anything with MAPQ < 20 and required multiple paired-end or split reads to support each breakpoint, and (ii) the XO and XX controls did not have nearly as many SV signals using the Smoove pipeline, showing that the widespread discordance is a property of the X’X sample alone (see Figures 2A and S1). Overall, up to 75% of the SV signals may involve transpositions and/or repeat-involved rearrangements unique to the X’, placing repeat sequences in contexts they are not found in on the X. That still leaves a minimum of 25% of SV signals involved strictly with “unique sequences” (not annotated as repeats) putatively arising from other structural rearrangements such as inversions.

| **SV Classification** | **Count on X** | **Count on X with one breakpoint located in a repeat** | **Count on X with both breakpoints located in a repeat** | **Count on X with one or both breakpoints located in repeats** | **Percent with one breakpoint located inside a repeat** | **Percent with both breakpoints located inside repeats** | **Percent with one or both breakpoints located inside repeats** |
| --- | --- | --- | --- | --- | --- | --- | --- |
| INV | 29 | 10 | 8 | 18 | 34.4828 | 27.5862 | 62.069 |
| DEL | 2697 | 987 | 1044 | 2031 | 36.5962 | 38.7097 | 75.3059 |
| DUP | 56 | 21 | 14 | 35 | 37.5 | 25 | 62.5 |
| TOTAL | 2782 | 1018 | 1066 | 2084 | 36.5924 | 38.3178 | 74.9101 |

**Supplementary table 7.** Classification of unmapped reads for each genotype.

| **Genotype** | **X0** | **XX** | **X’X** |
| --- | --- | --- | --- |
| **Mean proportion of unmapped reads (%)** | 6.46 | 3.71 | 4.76 |
| **Mean proportion of unmapped read pairs (neither mapped, %)** | 6.07 | 2.91 | 3.19 |
| **Read pairs classified as contaminants by Kraken2 (% of total reads)** | 2.93 | 1.30 | 1.44 |
| **Read pairs classified as bacterial by Kraken2 (% of total reads)** | 2.89 | 1.27 | 1.41 |
| **Read pairs classified as chordate by Kraken2 (% of total reads)** | 0.0029 | 0.0020 | 0.0017 |
| **Read pairs classified as other (viral, fungal, protozoan) by Kraken2 (% of total reads)** | 0.0058 | 0.0047 | 0.0044 |
| **Mean proportion of unmapped-unclassified pairs identified as contaminants by BLAST (% of total reads)** | 2.51 | 1.25 | 1.29 |
| **Read pairs identified as contamination by Kraken2 and BLAST (% of total reads)** | 5.44 | 2.54 | 2.73 |
| **Read pairs identified as GRC (% of total reads)** | 0.30 | 0.09 | 0.12 |
| **Read pairs identified as contamination or GRC (% of total reads)** | 5.73 | 2.64 | 2.85 |
| **Proportion of unmapped read pairs explained (%)** | 94.45 | 90.56 | 89.41 |

**Supplementary figure 1.** Structural variant calls from the XX genotype from Illumina 75bp paired-end short reads. These calls serve as an extra control along with calls from the X0 genotype, which, when constrasted with calls from the X’X genotype, provide further support that the X’ is enriched for complex rearrangements.

**Supplementary figure 2.** Expected (A) versus observed (B) differences in genomic coverage across the genome assembled *de novo* from PacBio reads from X’X individuals, indicating that the vast majority of reads from the X and X’ chromosomes collapsed together upon assembly. Note that the assembly is 291.63 Mb in length but appears shorter in the figure above because some scaffolds were shorter than the 20 Kb windows across which coverage was calculated, so such scaffolds were not included.

**Supplementary figure 3.** Distribution of contig lengths from the raw, short read SPAdes assembly of the X’. (A) All contigs; (B) contigs equal to or over 1Kb in length; (C) contigs less than 1Kb in length.

**Supplementary figure 4.** PacBio long reads from X’X individuals mapped to the X’X assembly. Now that the X and X’ inversion are separately assembled, PacBio reads map to the correct chromosomes with approximately expected coverage and could thus be used to fill some remaining gaps in the assembly of the X’ inversion.

**Supplementary figure 5.** Differential expression smear plot for X-linked genes with single-copy X’ homologs between the two types of females (XX versus X’X). Negative fold change (FC) represents upregulation of the gene copy in X’X females; positive FC represents upregulation of the gene in XX females. Transcript counts have been normalized such that overall X in X’X females expression equals that of XX females. Genes with significant fold change and significant adjusted P values are colored red.

**Supplementary references**

Altschul SF, Gish W, Miller W, Myers EW, Lipman DJ. 1990. Basic local alignment search tool. *J. Mol. Biol.* 215:403–410.

Anon. 2019. Picard toolkit. *Broad Inst. GitHub Repos.* [Internet]. Available from: https://broadinstitute.github.io/picard/

Blum M, Chang H-Y, Chuguransky S, Grego T, Kandasaamy S, Mitchell A, Nuka G, Paysan-Lafosse T, Qureshi M, Raj S, et al. 2020. The InterPro protein families and domains database: 20 years on. *Nucleic Acids Res.* 49:D344–D354.

Bray NL, Pimentel H, Melsted P, Pachter L. 2016. Near-optimal probabilistic RNA-seq quantification. *Nat. Biotechnol.* 34:525–527.

Breitwiese FP and Salzberg SL. 2016. Pavian: Interactive analysis of metagenomics data for microbiomics and pathogen identification. *bioRxiv*.

Uniprot Consortium. 2020. UniProt: the universal protein knowledgebase in 2021. *Nucleic Acids Res.* 49:D480–D489.

Uniprot Consortium. 2015. UniProt: a hub for protein information. *Nucleic Acids Res.* 43:D204–D212.

Crescente JM, Zavallo D, Helguera M, Vanzetti LS. 2018. MITE Tracker: an accurate approach to identify miniature inverted-repeat transposable elements in large genomes. *BMC Bioinformatics* 19:1–10.

Danecek P, Auton A, Abecasis G, Albers CA, Banks E, DePristo MA, Handsaker RE, Lunter G, Marth GT, Sherry ST, et al. 2011. The variant call format and VCFtools. *Bioinformatics* 27:2156–2158.

Darbo E, Herrmann C, Lecuit T, Thieffry D, van Helden J. 2013. Transcriptional and epigenetic signatures of zygotic genome activation during early *Drosophila* embryogenesis. *BMC Genomics* 14:226.

Dobin A, Davis CA, Schlesinger F, Drenkow J, Zaleski C, Jha S, Batut P, Chaisson M, Gingeras TR. 2013. STAR: ultrafast universal RNA-seq aligner. *Bioinformatics* 29:15–21.

Ellinghaus D, Kurtz S, Willhoeft U. 2008. LTRharvest, an efficient and flexible software for de novo detection of LTR retrotransposons. *BMC Bioinformatics* 9:1–14.

Flynn JM, Hubley R, Goubert C, Rosen J, Clark AG, Feschotte C, Smit AF. 2020. RepeatModeler2 for automated genomic discovery of transposable element families. *Proc. Natl. Acad. Sci.* 117:9451–9457.

Garrison E, Marth G. 2012. Haplotype-based variant detection from short-read sequencing. *ArXiv Prepr. ArXiv12073907*.

Gremme G, Steinbiss S, Kurtz S. 2013. GenomeTools: a comprehensive software library for efficient processing of structured genome annotations. *IEEE/ACM Trans. Comput. Biol. Bioinform.* 10:645–656.

Goday C, Esteban MR. 2001. Chromosome elimination in Sciarid flies. *Bioessays* 23:242-250.

Hodson CN, Jaron KS, Gerbi S, Ross L. 2022. Gene-rich germline-restricted chromosomes in black-winged fungus gnats evolved through hybridization. *PLOS Biol.* 20:e3001559.

Hu J, Zheng Y, Shang X. 2018. MiteFinderII: a novel tool to identify miniature inverted-repeat transposable elements hidden in eukaryotic genomes. *BMC Med. Genomics* 11:51–59.

Hungerford H. 1916. *Sciara* maggots injurious to potted plants. *J. Econ. Entomol.* 9:538–549.

Jones P, Binns D, Chang H-Y, Fraser M, Li W, McAnulla C, McWilliam H, Maslen J, Mitchell A, Nuka G, et al. 2014. InterProScan 5: genome-scale protein function classification. *Bioinformatics* 30:1236–1240.

Katumanyane A, Ferreira T, Malan AP. 2018. A Review of *Bradysia* spp. (Diptera: Sciaridae) as Pests in Nursery and Glasshouse Crops, With Special Reference to Biological Control Using Entomopathogenic Nematodes. *Afr. Entomol.* 26:1–13.

Kennedy MK. 1973. A culture method for *Bradysia impatiens* (Diptera: Sciaridae). *Ann. Entomol. Soc. Am.* 66:1163–1164.

Knight R, Maxwell P, Birmingham A. et al. PyCogent: a toolkit for making sense from sequence. *Genome Biol.* 8:R171.

Kokot M, Długosz M, Deorowicz S. 2017. KMC 3: counting and manipulating k-mer statistics. *Bioinformatics* 33:2759–2761.

Kriventseva EV, Kuznetsov D, Tegenfeldt F, Manni M, Dias R, Simão FA, Zdobnov EM. 2019. OrthoDB v10: sampling the diversity of animal, plant, fungal, protist, bacterial and viral genomes for evolutionary and functional annotations of orthologs. *Nucleic Acids Res.* 47:D807–D811.

Krueger F, Andrews SR. 2016. SNPsplit: Allele-specific splitting of alignments between genomes with known SNP genotypes. *F1000Research* 5.

Laetsch DR, Blaxter ML. 2017. BlobTools: Interrogation of genome assemblies. *F1000Research* 6:1287.

Langmead B, Salzberg SL. 2012. Fast gapped-read alignment with Bowtie 2. *Nat. Methods* 9:357–359.

Li H. 2013. Aligning sequence reads, clone sequences and assembly contigs with BWA-MEM. *ArXiv Prepr. ArXiv13033997*.

Li H, Handsaker B, Wysoker A, Fennell T, Ruan J, Homer N, Marth G, Abecasis G, Durbin R, 1000 Genome Project Data Processing Subgroup. 2009. The Sequence Alignment/Map format and SAMtools. *Bioinformatics* 25:2078–2079.

Li W, Yang Y, Xie W, Wu Q, Xu B, Wang Shaoli, Zhu X, Wang Shijun, Zhang Y. 2015. Effects of Temperature on the Age-Stage, Two-Sex Life Table of *Bradysia odoriphaga* (Diptera: Sciaridae). *J. Econ. Entomol.* 108:126–134.

Lu S, Wang J, Chitsaz F, Derbyshire MK, Geer RC, Gonzales NR, Gwadz M, Hurwitz DI, Marchler GH, Song JS, et al. 2020. CDD/SPARCLE: the conserved domain database in 2020. *Nucleic Acids Res.* 48:D265–D268.

Mansilla J, Pastoriza M, Pérez R. 2001. Study on biology and control of *Bradysia paupera* Tuomikoski (= *Bradysia difformis* Frey)(Diptera: Sciaridae). *Bol. Sanid. Veg. Plagas Esp.*

Mao H, Wang H. 2017. SINE_scan: an efficient tool to discover short interspersed nuclear elements (SINEs) in large-scale genomic datasets. *Bioinformatics* 33:743–745.

Marshall H, van Zweden JS, Van Geystelen A, Benaets K, Wäckers F, Mallon EB, Wenseleers T. 2020. Parent of origin gene expression in the bumblebee, *Bombus terrestris*, supports Haig’s kinship theory for the evolution of genomic imprinting. *Evol. Lett.* 4:479–490.

Metz, CW. 1925. Chromosomes and Sex in *Sciara*. *Science* 61:212–214.

Nigro RG, Campos MCC, Perondini ALP. 2007. Temperature and the progeny sex-ratio in Sciara ocellaris (Diptera, Sciaridae). *Genet. Mol. Biol.* 30:152–158.

O’Leary NA, Wright MW, Brister JR, Ciufo S, Haddad D, McVeigh R, Rajput B, Robbertse B, Smith-White B, Ako-Adjei D, et al. 2015. Reference sequence (RefSeq) database at NCBI: current status, taxonomic expansion, and functional annotation. *Nucleic Acids Res.* 44:D733–D745.

Pruitt KD, Tatusova T, Maglott DR. 2007. NCBI reference sequences (RefSeq): a curated non-redundant sequence database of genomes, transcripts and proteins. *Nucleic Acids Res.* 35:D61–D65.

Quinlan AR, Hall IM. 2010. BEDTools: a flexible suite of utilities for comparing genomic features. *Bioinformatics* 26:841–842.

R Core Team. 2022. R: A Language and Environment for Statistical Computing. Vienna, Austria: R Foundation for Statistical Computing Available from: https://www.R-project.org/

Rieffel S M, Crouse HV. 1966. The elimination and differentiation of chromosomes in the germ line of *Sciara*. *Chromosoma* 19:231–276.

Riehl K, Riccio C, Miska EA, Hemberg M. 2022. TransposonUltimate: software for transposon classification, annotation and detection. *Nucleic Acids Res.* 50:e64–e64.

Robinson MD, McCarthy DJ, Smyth GK. 2010. edgeR: a Bioconductor package for differential expression analysis of digital gene expression data. *Bioinformatics* 26:139–140.

Ruan J, Li H. 2020. Fast and accurate long-read assembly with wtdbg2. *Nat. Methods* 17:155–158.

de Saint Phalle B, Sullivan W. 1996. Incomplete sister chromatid separation is the mechanism of programmed chromosome elimination during early *Sciara coprophila* embryogenesis. *Development* 122:3775–3784.

dos Santos G, Schroeder AJ, Goodman JL, Strelets VB, Crosby MA, Thurmond J, Emmert DB, Gelbart WM, Consortium the F. 2014. FlyBase: introduction of the *Drosophila melanogaster* Release 6 reference genome assembly and large-scale migration of genome annotations. *Nucleic Acids Res.* 43:D690–D697.

Seppey M, Manni M, Zdobnov EM. 2019. BUSCO: Assessing Genome Assembly and Annotation Completeness. In: Kollmar M, editor. Gene Prediction: Methods and Protocols. New York, NY: Springer New York. p. 227–245. Available from: https://doi.org/10.1007/978-1-4939-9173-0_14

Smit A, Hubley R, Green P. 2015. RepeatMasker Open-4.0. 2013–2015.

Smith-Stocking H. 1936. Genetic studies on selective segregation of chromosomes in *Sciara coprophila* Lintner. *Genetics* 21:421–443.

Starostina E, Tamazian G, Dobrynin P, O’Brien S, Komissarov A. 2015. Cookiecutter: a tool for kmer-based read filtering and extraction. *bioRxiv*:024679.

Urban J, Bashir A, Sebra R, Foulk M, Howison M, Gerbi S. 2015. Re-replication Origins in *Sciara* DNA Puffs Revealed by New and Old Genomic Technologies including Nanopore Sequencing. *FASEB J.* 29:561–569.

Urban JM, Bateman JR, Garza KR, Borden J, Jain J, Brown A, Thach BJ, Bliss JE, Gerbi SA. 2021a. *Sciara coprophila* larvae upregulate DNA repair pathways and downregulate developmental regulators in response to ionizing radiation. Genomics Available from: http://biorxiv.org/lookup/doi/10.1101/2021.10.28.466123

Urban JM, Foulk MS, Bliss JE, Coleman CM, Lu N, Mazloom R, Brown SJ, Spradling AC, Gerbi SA. 2021b. High contiguity de novo genome assembly and DNA modification analyses for the fungus fly, *Sciara coprophila*, using single-molecule sequencing. *BMC Genomics* 22:643.

Vaser R, Sović I, Nagarajan N, Šikić M. 2017. Fast and accurate de novo genome assembly from long uncorrected reads. *Genome Res.* 27:737–746.

Villanueva-Sánchez E, Ibáñez-Bernal S, Lomelí-Flores J, Valdez-Carrasco J. 2013. Identificación y caracterización de la mosca en el cultivo de nochebuena (*Euphorbia pulcherrima*) en el centro de México. *Acta Zool. Mex.* 29:363–375.

Wickham H. 2016. Data analysis. In: ggplot2. Springer. p. 189–201.

Wood DE, Lu J, Langmead B. 2019. Improved metagenomic analysis with Kraken 2. *Genome Biol* 20:257.

Xiong W, He L, Lai J, Dooner HK, Du C. 2014. HelitronScanner uncovers a large overlooked cache of Helitron transposons in many plant genomes. *Proc. Natl. Acad. Sci.* 111:10263–10268.
